# How to Assess Scar Quality in Pediatric Burn Patients: A Systematic Review on the Type and Content of Outcome Measurement Instruments

**DOI:** 10.1093/jbcr/iraf048

**Published:** 2025-05-03

**Authors:** Frederique M Kemme, Adinda Mieras, Johannes C F Ket, Annebeth Meij-de Vries, Paul P M van Zuijlen, Anouk Pijpe

**Affiliations:** Alliance of Dutch Burn Care (ADBC), Burn Center, Red Cross Hospital, Beverwijk, The Netherlands; Department of Plastic Reconstructive and Hand Surgery, Amsterdam UMC Location Vrije Universiteit van Amsterdam, Amsterdam, The Netherlands; Amsterdam Movement Sciences, Tissue Function and Regeneration, Amsterdam UMC, Amsterdam, The Netherlands; Alliance of Dutch Burn Care (ADBC), Burn Center, Red Cross Hospital, Beverwijk, The Netherlands; Department of Plastic Reconstructive and Hand Surgery, Amsterdam UMC Location Vrije Universiteit van Amsterdam, Amsterdam, The Netherlands; Amsterdam Movement Sciences, Tissue Function and Regeneration, Amsterdam UMC, Amsterdam, The Netherlands; Medical Library, Vrije Universiteit, Amsterdam, The Netherlands; Alliance of Dutch Burn Care (ADBC), Burn Center, Red Cross Hospital, Beverwijk, The Netherlands; Department of Surgery, Red Cross Hospital, Beverwijk, The Netherlands; Paediatric Surgical Center, Amsterdam UMC Location University of Amsterdam, Emma Children’s Hospital, Amsterdam, The Netherlands; Alliance of Dutch Burn Care (ADBC), Burn Center, Red Cross Hospital, Beverwijk, The Netherlands; Department of Plastic Reconstructive and Hand Surgery, Amsterdam UMC Location Vrije Universiteit van Amsterdam, Amsterdam, The Netherlands; Amsterdam Movement Sciences, Tissue Function and Regeneration, Amsterdam UMC, Amsterdam, The Netherlands; Paediatric Surgical Center, Amsterdam UMC Location University of Amsterdam, Emma Children’s Hospital, Amsterdam, The Netherlands; Department of Plastic Reconstructive and Hand Surgery, Red Cross Hospital, Beverwijk, The Netherlands; Alliance of Dutch Burn Care (ADBC), Burn Center, Red Cross Hospital, Beverwijk, The Netherlands; Department of Plastic Reconstructive and Hand Surgery, Amsterdam UMC Location Vrije Universiteit van Amsterdam, Amsterdam, The Netherlands; Amsterdam Movement Sciences, Tissue Function and Regeneration, Amsterdam UMC, Amsterdam, The Netherlands

**Keywords:** scar quality, PROM, CROM, measurement instrument, burns, pediatrics

## Abstract

Measuring scar quality is important for monitoring scar development and evaluating treatment outcomes. Given the substantial representation of children within the burn population and their susceptibility to lifelong scarring, evaluating scar quality is particularly important in this group. This study provides an overview of outcome measurement instruments used to assess scar quality in pediatric burn patients. A systematic literature search was conducted in PubMed, EMBASE and Web of Science covering studies published up to March 25, 2024. We included original research studies in English that measured at least one scar quality characteristic in pediatric burn patients. We included 328 studies and identified 585 outcome measurement instruments: clinician-reported outcome measures (CROMs) (53%), measurement devices (25%), and patient-reported outcome measures (PROMs) (22%). The most frequently used instruments were the (modified) Vancouver Scar Scale, ultrasound, and the Patient and Observer Scar Assessment Scale Patient scale, respectively. Thickness and itch were the most frequently assessed scar characteristics. The use of PROMs has increased over the past decade, particularly after 2016, highlighting their growing attention. Among the studies using PROMs, 42% reported age-related conditions, with thresholds for independent completion ranging from 5 to 16 years. However, CROMs are the most frequently used instruments. While PROMs, CROMs and measurement devices are valuable, they are often not specifically designed for or validated in pediatric burn patients, and therefore they could benefit from further development or validation to better address the specific needs of pediatric burn patients.

## INTRODUCTION

Children represent a large proportion of the burn population globally.^1^ The transitional phases of childhood and adolescence can be a challenging time, as most young individuals experience physical and psychological changes. Children with severe burns are at risk for lifelong scarring, which can cause several cosmetic and functional problems and a lower health-related quality of life, compared to a normative population.^2–4^ Measuring scar quality, defined as the visual, tactile and sensory characteristics of a burn scar, is therefore of particular importance in this group.^5^ Standardized assessment of scar quality helps both patients, caregivers, and healthcare providers to determine whether any treatment is necessary, supports shared decision-making, and aids in evaluating the effect of therapeutic interventions.^5^ Additionally, it serves as a valuable tool to monitor scars over time and it helps patients understand the future perspective of their scar.^6^

A considerable number of outcome measurement instruments are available for measuring scar quality, including objective and subjective tools.^6,7^ Patient-Reported Outcome Measures (PROMs) and Clinician-Reported Outcome Measures (CROMs) are subjective tools based on an individual’s evaluation of scar quality, with PROMs particularly advantageous for assessing aspects of the scar that experienced by the patient (eg, pain and itch), whereas CROMs provide an evaluation from the perspective of a clinician or other healthcare professional.^7^ Objective tools provide quantitative assessment of a scar and include measurement devices measuring specific scar characteristics like color, pliability or thickness.^8–14^ Choosing the appropriate outcome measure requires careful consideration of its alignment with the purpose (in this case; measuring scar quality), type of patient population, feasibility of the scale, as well as its validity and reliability.^15,16^ In children, measuring outcomes requires additional considerations, such as a child-friendly environment, age-appropriate communication, age-related interests, parental involvement and time management. Additionally, PROMs for children should be tailored to their developmental stages and should be explicitly tested for children’s understanding of health-related concepts and terminology. Moreover, given the significant role of parental involvement in pediatric assessments, it is essential that PROMs for children accommodate the use of proxy reports, as many children may be too young or unable to self-report their experiences.^3,17^

While a few outcome measurement instruments have been developed for or validated in pediatric patients, none were specifically developed to primarily assess scar quality. Instruments such as the Brisbane Burn Scar Impact Profile (BBSIP), the Children’s Burn Outcome Questionnaire (BOQ) and the Bock Questionnaire focus on Health Related Quality of Life (HRQoL) and functional outcomes.^18–20^ Furthermore, the BBSIP and BOQ are burn-specific, whereas the Bock is not.^18^ Although these instruments include some items related to the construct scar quality, they were not primarily developed for this purpose or with involvement of the target population, and therefore may not capture all relevant aspects of scar quality.

There is currently a lack of a comprehensive overview of scar quality measurement instruments and their content used in children after burns. Therefore, we performed a systematic review, providing clinicians and researchers with insights and guidance on the presently available most appropriate tools for assessing scar quality in pediatric burn patients.

## METHODS

The protocol was registered in the International Prospective Register of Systematic Reviews (PROSPERO) on 07/10/2023 (ID: CRD42023459769). During the process of the review, we learned that our initial search was too narrow, as many additional relevant studies were found through reference checking. Therefore, we performed a broader second search, omitting 2 search blocks, namely 'assessment/tool' and ‘quality/outcome’ to capture a wider range of studies. For this reason, the first submission and registration date does not entirely match the final study procedure. This systematic review was reported following the Preferred Reporting Items for Systematic Reviews and Meta-analyses (PRISMA) statement (www.prisma-statement.org).

### Search strategy

A literature search was performed in PubMed, EMBASE, and Web of Science up to March 25, 2024. The search was based on the search used in 2 previous systematic reviews conducted by our research group and it was further refined with support of a medical information specialist (J.C.F.K.).^5,21^ The full search strategies with the search dates are provided in Supplementary File 1. The search included controlled terms and free text terms (including synonyms and closely related words) for “scars” and “children” and “burns.” DedupEndNote (http://dedupendnote.nl/) was used for deduplication.

### Study selection

The screening procedure was conducted with the use of the systematic review web app Rayyan (www.rayyan.ai). All titles, abstracts and full texts were screened independently by 2 reviewers (F.M.K. and A.M.) to select relevant studies. Disagreements between reviewers were discussed and resolved through discussion with a third investigator (A.P.) if needed. The following criteria were applied to select relevant studies:

#### Study types and language

Original research studies written in English were included. Qualitative studies in humans were only included if an actual assessment of scar quality took place, rather than only gathering subjective experiences without an outcome measurement tool. Conference abstracts, protocols, opinion papers, and systematic reviews were excluded, as well as studies that mentioned a scar characteristic without specifying how this characteristic was quantified. Animal studies and studies using invasive methods or reporting on biological characteristics of scar quality alone (eg, obtained by histology) were excluded, as these methods are not applicable in clinical practice.

#### Population

Studies concerning children (<18 years old) with a burn scar were included. For studies with a mixed population (children and adults), and for studies including scars resulting from burns as well as other causes, studies were included if the methods section indicated the intention to apply the measurement instrument in the whole study population (ie, if children with burn scars were intended to be included in the study), or if it was specifically described which measurement instrument was used in children. If multiple studies were published based on the same study population (eg, with follow-up results at 3 months and 12 months), only the most recent article was included in this review.

#### Outcome measurement instruments

Studies were included if they described the use and content of an outcome measurement instruments (ie, questionnaires, measurement devices or scar scales) that measured at least one specific characteristic of scar quality in children with burn scars, and defined how these characteristics were assessed. Any scar characteristics measured in a flap after reconstructive surgery, and not in the burn scar, were excluded. We excluded questionnaires primarily designed to measure health-related quality of life (eg, BOQ, BSHS, EQ-5D) rather than scar quality.^22–24^ Health-related quality of life questionnaires containing a subscale on scar characteristics, such as the BBSIP, were included with only the scar quality-specific data.^25^ Measurement instruments reporting on physical (eg, range of motion) and psychosocial functioning, as well as items as “satisfaction with treatment” were excluded. Studies that described the use of their own developed (unestablished, invalidated) outcome measure, for example a self-invented questionnaire, to evaluate at least 1 item of scar quality, were included as well.

As the aim of this study was to identify the type and content of all instruments used for measuring scar quality in children after burns, we did not evaluate the quality of the included studies.

### Data extraction

Data was extracted using a standardized data extraction form in Excel and included the following parameters: (1) study design, year, origin of the study (country), (2) characteristics of the study sample: sample size, age of the participants, cause of the burn, and (3) characteristics of the reported outcome measurement instrument(s): type of instrument, description of the construct measured, number of items concerning scar quality, and the content of these items. Countries were categorized based on country and geographical regions: Europe, North America, South America, Asia, Africa, and Oceania. Data extraction was performed independently by F.M.K and A.M. Discrepancies between them were minimal, with <5% of the extracted parameters differing, and were resolved through discussion.

All identified measurement instruments were categorized based on their type: CROM, PROM, or measurement device. Unnamed scales were categorized under “unlabeled.” Scar scales were classified as modified versions of the original if any items were added, removed, or rephrased, or if there were any changes to the response options or answering categories. Only items specifically referring to scar quality were extracted from included studies. A single overall opinion score about the scar was also included.

Items with closely related constructs were grouped under broader categories to facilitate consistent reporting and analysis. For example, thickness, elevation, and height were grouped together as “thickness”, as they all reflect the physical dimension of the scar in terms of vertical extension. Similarly, stiffness, elasticity, and pliability were combined as “stiffness”, as they represent related mechanical properties of the scar tissue. Vascularity was used as an umbrella term for redness or vascularity. These groupings were based on consensus among the authors and a review of definitions in the included studies.

Patient-reported outcome measures were divided into those reported by the child and those reported by proxy (parent/caregiver). All PROMs were considered patient-reported and thus child-reported unless it was specifically stated that they were reported by proxy. Proxy-reported PROMs were reported separately. If the PROMs were completed with parental support, or if specific age-related guidelines were applied, these were documented accordingly. If subjective items such as pain and itch were included and it was not explicitly stated that these were scored by the observer, they were considered as patient-reported items.

## RESULTS

### Study selection

**Figure 1.**
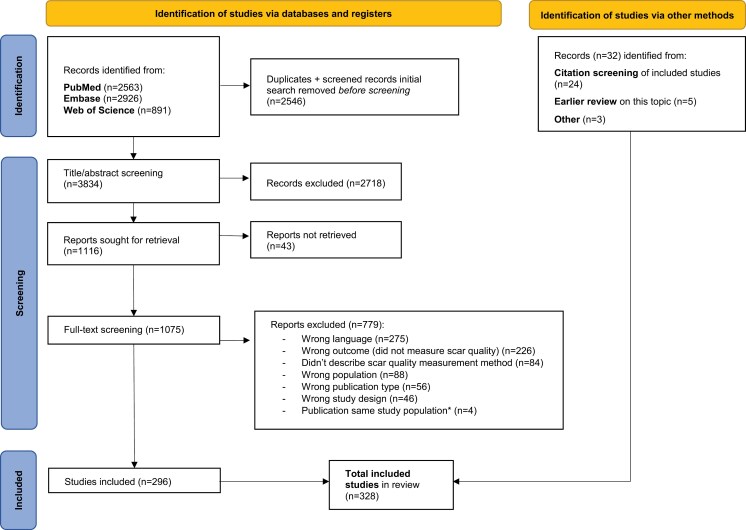
PRISMA Flow Chart

A total of 6380 records were identified with this systematic literature search across PubMed (n = 2563), EMBASE (*n* = 2926) and Web of Science (*n* = 891) up to March 25, 2024. After deduplication, 3834 records remained. The title/abstract screening resulted in the selection of 1075 records for full-text screening. Ultimately, 296 studies were included for data extraction. An additional 32 studies were included through reference checks and other sources, bringing the total number of included studies to 328. The study selection process is shown in Figure 1.

### Study and outcome measurement instrument characteristics

The characteristics of the included studies and outcome measurement instruments are summarized in Table 1. All studies were published between 1978 and 2024. Most studies originated from the United States (*n* = 60, 10%), followed by China (*n* = 43, 7%) and Australia (*n* = 25, 4%). In the majority (92%) of the studies, the outcome measurement instruments were applied to assess scar quality in a clinical context, rather than as part of clinimetric validation or development studies. In 41% of the studies, the study population consisted exclusively of children. A total of 43 different outcome measurement instruments were identified, as detailed in Supplementary Digital Content 2a,b,d. In these tables, blue labels indicate outcome measurement instruments that are either widely used in research and clinical practice (“well-established”) or have been validated in the target population. Modified or self-created outcome measures are not highlighted. Measurement devices in Supplementary Digital Content 2d were categorized based on their type of technology. When including all self-created instruments, counting all measurement devices individually, and treating modified versions as unique instruments, the total number of identified outcome measurement tools increases to 127. The mean number of outcome measurement instruments used was 2 per study (range 1-10, median 1). Clinician-Reported Outcome Measures were most frequently used (53%), followed by measurement devices (25%) and PROMs (22%).

**Table 1.** Characteristics of Included Studies and Outcome Measurement Instruments^a^

Included studies	*N* = 328 (100%)	
**Study design**		
Clinical	301	(92%)
Clinimetric	22	(7%)
Developmental	5	(1%)
**Study population**		
Children + adults	194	(59%)
Exclusively children	134	(41%)
**Study origin**		
Asia	103	(31%)
Europe	94	(29%)
North America	76	(23%)
Oceania	27	(8%)
Africa	19	(6%)
South-America	6	(2%)
**Total number of identified outcome measurement instruments**	***N* = 585 (100%)**	
*Type and order of most frequently used outcome measurement instruments*		
Clinician-reported outcome measures	308 (53%)	
Measurement devices	148 (25%)	
Patient-reported outcome measures	129 (22%)	

^a^Percentages may not sum up to 100% due to rounding differences.

### Clinician-reported outcome measurement instruments

We identified 51 different (versions of) named CROMs, displayed in Supplementary Digital Content 2a. Taking all versions into account, the Vancouver Scar Scale (VSS) was the most frequently used CROM (171 out of 308, 56%), followed by the Observer scale of the Patient and Observer Scar Assessment Scale (POSAS, all versions) (55 out of 308, 18%). No differences were observed in the frequency of use of both scales worldwide, and they were equally well represented across all continents. The VSS was most frequently modified; the original version by Sullivan was used most often (54 times).^26^ In twelve studies, the (m)VSS was partially used as a PROM, despite being originally developed as a CROM. The modified version by Nedelec et al. was most frequently used.^27^ Clinicians most frequently assessed the items vascularity and thickness, while patients mostly frequently assessed itch, followed by pain.

Apart from the 51 named CROMs, we also identified 61 unnamed scales. Overall, the CROMs used a median of 4 items (range 1-9) to assess scar quality. Thickness, pigmentation, and vascularity were the most frequently assessed items (Table 2).

**Table 2.** Most Frequently Assessed Items per Type of Outcome Measure

	CROM	PROM	Measurement device
**First**	Thickness	Itch	Thickness
**Second**	Pigmentation	Pain	Stiffness
**Third**	Vascularity	Color and thickness	Erythema

### Patient-reported outcome measurement instruments

A total of 20 different (versions) of patient-reported outcome measurement instruments were identified, displayed in Supplementary Digital Content 2b. The first study describing the use of a PROM to assess scar quality in children was first documented in 1985.^28^ Over the years, the number of studies using PROMs remained limited to 1-5 studies annually. Since around 2016, the use of PROMs has grown to 10 or more studies published annually. A total of 24 unnamed scales were found.

The identified PROMs assessed a median of 3 scar quality items, most commonly itch and pain, followed by color and thickness. The POSAS Patient scale (v1.0: 21 studies; v2.0: 34 studies) was the most frequently used patient-reported scale (55 out of 129, 43%). The BBSIP was the second most frequently used (6 out of 129, 5%) and the University of North Carolina “4P” Scar Scale (UNC-4P) scale the third (4 out of 129, 3%).

Any specific (age-related) conditions for filling out the PROM were described in 52 out of 129 studies (42%) and are displayed in Supplementary Digital Content 2c. These conditions were most frequently reported for the POSAS Patient scale (32 out of 52 studies using POSAS Patient scale, 62%) and demonstrated variability in the age at which children could independently complete a PROM, ranging from 5 to 16 years.

### Measurement devices

We found 44 different measurement devices, of which the ultrasound was the most frequently used device (29 times), followed by the Cutometer (16 times) and the Dermaspectrometer (12 times) (Supplementary Digital Content 2d). Ultrasounds used included a variety of models, such as high-frequency probes (eg, 10-MHz B-mode ultrasound, 6-18 MHz linear-array transducers) and systems designed for precise tissue imaging, such as the GE Healthcare Venue 40 MSK ultrasound and the Siemens CUSON S2000 with Virtual Touch Tissue Imaging and Quantification (VTIQ). All Cutometers (except for one unknown brand) used in the studies were by Courage+Khazaka Electronic GmbH, with specific models including the SEM 474, MPA 580, and EM 5801. The Dermaspectrometer manufactured by Cortex Technology (Hadshund, Denmark) (version not reported) was the most frequently used. Most (76%) of the measurement devices measured a single item, with a maximum of 5 items being measured by a single device. Following the order of the most frequently used devices, thickness, stiffness, and erythema were the most frequently measured items.

## DISCUSSION

This systematic review provides a comprehensive overview of the outcome measurement instruments and their content used to assess scar quality in pediatric burn patients. Based on studies published between 1978 and 2024, scar quality in children with burn scars was most frequently measured using CROMs, followed by measurement devices and PROMs. The (m)VSS, the ultrasound, and the POSAS Patient scale were used the most, respectively. The most frequently scar items assessed were thickness and itch. The reliability and applicability of the currently used outcome measurement instruments are limited or have not been adequately assessed in the pediatric population. None of these instruments have been specifically developed for or validated in children, which may impact their effectiveness in this population. A significant challenge in pediatric settings is determining the appropriate age at which children can reliably self-report.

Both the order of most frequently used instruments and scar characteristics assessed, show similarities with measuring scar quality across a broader population.^5^ This may reflect a consistent approach among clinicians in assessing scar quality across different age groups. However, it may also indicate a gap due to the lack of a child-specific instrument for measuring scar quality, which could address aspects overlooked by the abovementioned outcome measurement instruments. A notable finding is the discrepancy in the items assessed by CROMs compared to PROMs. Clinicians primarily focus on measurable and visible characteristics, such as thickness and pigmentation, whereas patients tend to prioritize subjective experiences, such as itch and pain. Clinicians should be aware of these differing priorities, and this underscores the importance of incorporating patient perspectives in the development of outcome measurement instruments.

Additionally, our review identified a considerable number of unlabeled measurement instruments, which are essentially self-developed tools that lack formal validation. These tools may reflect either a lack of awareness regarding existing validated instruments or a conscious decision to create a new instrument tailored to specific needs. However, the use of such unvalidated instruments limits the comparability of results across studies and hinders international standardization. To improve the consistency and reliability of scar quality assessments in pediatric burn patients, it is essential to prioritize the use of validated instruments.

Each category of outcome measurement instruments serves distinct yet complementary roles in scar quality assessment. Clinician-reported outcome measures provide the perspective of a clinician on observable aspects of the scar, which can be valuable for clinical decision making. The VSS by Sullivan et al. was the most frequently used CROM according to this review. After its development in 1990, it widely became the most frequently used scar scale.^7^ A reasonable explanation for our findings seems that the VSS has been established for the longest time, gaining recognition among clinicians, which likely contributed to its prevalence in clinical practice and research settings. However, while widely used, the validity, reliability, and feasibility of the VSS in pediatric burn patients remain understudied. Moreover, the development of a number of modified versions over time has led to inconsistencies in its application. Although the original authors laid a strong foundation, subsequent adaptations were often made without a structured validation process or clear methodological justification. Notably, the original authors themselves acknowledged in their discussion that the scale was not in its final form and suggested modifications, including the importance of patient-reported symptoms such as pain and itch. However, the absence of centralized oversight in the development of these versions has resulted in variations that may compromise comparability across studies. Interestingly, in our study, the (m)VSS was also used as a combined clinician- and patient-reported scale, despite the fact that we found no evidence that support the use of the (m)VSS as such in our extensive search. However, this illustrates an attempt to incorporate patient perspectives within CROMs, yet such scales do not fully align with the principles of PROMs and underscore the need for distinct tools to capture the child’s voice comprehensively. Furthermore, achieving international consensus on the essential items to be included in CROMs could enhance their relevance and applicability. A previous international Delphi study for POSAS 3.0 has already established a consensus on the definition of “scar quality”, involving international scar experts who reached agreement on 9 essential items for assessing scar quality. This is an important step towards a unified approach in scar quality assessment, providing a foundation for further refinement of these outcome measurements.^29^

On the other hand, PROMs aim to capture the patient’s own perceptions, as they are based on the idea that individuals are the most reliable observers of their own health status and experiences.^17^ Patient-Reported Outcome Measures have gained attention in general healthcare, but also specifically in terms of scar assessment, as seen by the growing number of scar-specific patient-reported scales, especially since 2016.^5,30–35^ We found the POSAS Patient scale as the most frequently used PROM in children with burn scars. In addition to symptoms such as pain and itch, in 2004, the POSAS was the first scale to incorporate the patient’s opinion on visual and physical scar characteristics.^7^ Most studies used version 2.0 of the POSAS; no publications on version 3.0 were identified, as this version has only recently been released (2023).^36^ Although the POSAS was developed with input from patients, it was not specifically developed for children, nor has it been validated for its use in children. The BBSIP, which was the second most used PROM according to our study, was developed in 2015 to primarily measure health-related quality of life in children with burn scars, but it includes subscales on visual and physical scar characteristics and symptoms, specifically in the “feelings” and “what scars are like” sections of the instrument.^34^

Measurement devices provide objective quantitative data on scar characteristics (such as color, pliability, and thickness), thereby enhancing the understanding of scar quality from a clinical perspective. However, these tools often require specialized training and can be limited by their accessibility and costs.^7^ Additionally, none of the currently available devices perfectly balance clinical relevance with feasibility, making it challenging to adopt a single device universally.^18^ When it comes to pediatric populations, the use of these measurement devices presents additional challenges. Children’s anatomical and physiological differences, such as skin thickness and elasticity, can affect the accuracy and applicability of the measurements obtained. Moreover, the need for child-friendly approaches in both device design and data collection are essential to ensure comfort and cooperation during assessments. Previous studies highlight that a limitation of subjective scar scales is that their accuracy depends on the assessor’s experience, scar severity, and scar age, and therefore recommend a combination of objective scar assessment tools with subjective scales to assess multiple scar characteristics simultaneously.^37,38^ For all the aforementioned categories—CROMs, PROMs, and measurement devices—it is essential that these are specifically validated for use in children to ensure their relevance, feasibility and accuracy in this population.

Importantly, the use of outcome measurement instruments for assessing scar quality in pediatric burn patients must be contextualized within the setting in which they are employed. Most of the literature on scar assessment originates from middle to high-income countries, while there remains a significant group of severely burned children in low-income countries where measuring scar quality may not be feasible due to financial reasons, training requirements, and availability of the equipment.

Our review showed that, despite the increased use of PROMs to assess scar quality in pediatric patients since 2016, use of PROMs remains limited compared to CROMs, which is also evident in scar assessment among a broader population.^5^ Although PROMs are designed to be completed by patients themselves, in the pediatric context, they are often filled out by proxies (usually parents or caregivers). An important finding of this review is the wide range observed in the age at which children are considered able to fill out a PROM independently. While literature suggest that children from around the age of 8 years old can reliably report about their health, the absence of clear guidelines for pediatric use of outcome measurement instruments present a barrier.^17,39,40^ Proxy-reports are still advisable under some circumstances, for example, for very young or intellectually disabled children who may lack the cognitive and communicative abilities to self-report accurately.^17^ While this is unavoidable for these children, it is important to recognize that discrepancies between parent and child reports can be significant, as evidenced by Meyer et al., who found that children rated their scars more positively than their parents did.^41^ Wright and Fulwiler emphasized that parents, especially mothers, might have biased perceptions due to emotional impacts, further underscoring the need for direct child input.^42^ Moreover, certain scar characteristics, such as pain and itch, pose particular challenges for proxy reporting, as it can be difficult to accurately convey the child’s experience of these sensations. A key aspect of proxy versions is the ability to distinguish between the caregiver’s own perspective and their interpretation of the child’s perspective.^43,44^ For example, the EQ-5D-Y-3L offers a tailored approach by including 3 versions: a self-report for children aged 8-15, a proxy-proxy version where caregivers provide their own assessment, and a proxy-patient version where caregivers estimate the child’s perspective.^45^ Parent reports differ from those of children and are nonetheless valuable but should not be used to discount or replace the views of children who are able to report on their own behalf.^17,39,46–49^ This highlights the importance of incorporating the child’s viewpoint in assessments to ensure a good understanding of scar quality. Moreover, the observed discrepancies between parent and child assessments suggest the need for further research into the differences in their assessments of burn scar quality.

Additionally, the limited use of PROMs in pediatric burn patients may be attributed to the lack of appropriate, validated PROMs, specifically developed with input from and for children with burn scars. As a result, PROMs originally designed for adults (and/or not *specifically* for and with children), are used in children, which might be less suitable in this population due to their terminology and are unlikely to adequately capture the unique experiences and developmental stages of children.^17,18^ Although the BBISP, the second most used PROM till date in our study, was developed with and for children with burns and contains some items on scar quality, it is intended to measure quality of life and the development did not fully capture all relevant items on scar quality.^34^ Given the limitations of existing PROMs, alternative approaches have been explored to allow children to self-report their experiences. For instance, Morris et al. developed an itch assessment scale (the Itch Man Scale) specifically for and with pediatric burn patients, demonstrating that tailored tools can effectively capture the subjective experiences of children.^50^ The limited use of PROMs and the reliance on proxies in pediatric settings suggests a potential gap in capturing the child’s perspective on scar quality.

One of the strengths of this review was the comprehensive, extensive, and adaptive search strategy performed in 3 databases: PubMed, EMBASE, and Web of Science. Following this search, the reference lists of the included studies were also screened for relevant studies. This approach reduced the risk of missing important studies. However, several limitations should be noted. A limitation of our study was the challenge of determining which outcome measurement instruments to include when their primary focus was not scar quality but contained at least a subscale addressing this domain. For example, we excluded instruments primarily designed to measure health-related quality of life, such as the BOQ, BSHS, and EQ-5D, but included the BBISP, which, despite its broader focus on quality of life, includes subscales with scar quality items. This selection could have led to the capture of a broader range of instruments relevant to scar assessment. Additionally, several important limitations exist regarding the studies included in this review. First, only a small proportion of the included studies exclusively focused on children, which may affect the generalizability of our findings. Second, the limited reporting of any (age-related) when using PROMs restricts the applicability of our findings to specific age groups. Third, a considerable number of excluded studies (84 out of 779, 11%) did not clearly report how scar quality was measured. Consequently, this study or the instrument could not be included as it lacked sufficient clarity on its measurement approach.

## CONCLUSION

This review highlights that at this moment, CROMs are the most frequently used tools for assessing scar quality in pediatric burn patients, followed by measurement devices, and PROMs. All outcome measurement instruments will benefit from further development or validation to better address the specific needs of pediatric burn patients, as many currently used tools are not specifically developed or validated for use in this population. There is a lack of an age-appropriate, validated PROM for pediatric burn patients. Active involvement of the target population is essential in this development process. A more uniform approach to scar quality measurements is crucial for improving consistency and comparability across different settings. By addressing these gaps, we can enhance scar quality assessment and burn and scar management in children.

## Supplementary Material

iraf048_suppl_Supplementary_File_S1

iraf048_suppl_Supplementary_Material
