# Supplementary material for: How to Assess Scar Quality in Pediatric Burn Patients: A Systematic Review on the Type and Content of Outcome Measurement Instruments
Source: J Burn Care Res. 2025 May 3;46(6):1294–301. doi: 10.1093/jbcr/iraf048 (PMC12596693; doi:10.1093/jbcr/iraf048)
Supplement: iraf048_suppl_Supplementary_File_S1 [file iraf048_suppl_supplementary_file_s1.docx]

**Supplementary File 1: full search strategies**

**Search strategy for PubMed (27 March 2024)**

| **Search** | **Query** | **Results** |
| --- | --- | --- |
| **#4** | #1 AND #2 AND #3 | **2,563** |
| **#3** | "Cicatrix"[Mesh] OR "cicatri*"[tiab] OR "scar"[tiab] OR "scars"[tiab] OR "scarring"[tiab] OR "keloid*"[tiab] | **119,374** |
| **#2** | "Burns"[Mesh:NoExp] OR "Burns, Chemical"[Mesh] OR "burn"[tiab] OR "burns"[tiab] OR "scald*"[tiab] OR "postburn*"[tiab] OR ("thermal"[tiab] AND "injur*"[tiab]) | **93,109** |
| **#1** | **"**infan*"[tw] OR "child*"[tw] OR "adolescen*"[tw] OR "pediatric*"[tw] OR "paediatric*"[tw] OR "pube*"[tw] OR "juvenil*"[tw] OR "school*"[tw] OR "newborn*"[tiab] OR "new-born*"[tiab] OR "neo-nat*"[tiab] OR "neonat*"[tiab] OR "premature*"[tiab] OR "postmature*"[tiab] OR "pre-mature*"[tiab] OR "post-mature*"[tiab] OR "preterm*"[tiab] OR "pre-term*"[tiab] OR "baby"[tiab] OR "babies"[tiab] OR "toddler*"[tiab] OR "youngster*"[tiab] OR "preschool*"[tiab] OR "kindergart*"[tiab] OR "kid"[tiab] OR "kids"[tiab] OR "playgroup*"[tiab] OR "play-group*"[tiab] OR "playschool*"[tiab] OR "prepube*"[tiab] OR "preadolescen*"[tiab] OR "junior high*"[tiab] OR "highschool*"[tiab] OR "senior high"[tiab] OR "young people*"[tiab] OR "minors"[tiab] | **5,220,289** |

**Search strategy for Embase.com (27 March 2024)**

| **Search** | **Query** | **Results** |
| --- | --- | --- |
| **#4** | #1 AND #2 AND #3 NOT 'conference abstract'/it | **2,926** |
| **#3** | 'scar'/exp OR 'cicatri*':ti,ab,kw OR 'scar':ti,ab,kw OR 'scars':ti,ab,kw OR 'scarring':ti,ab,kw OR 'keloid*':ti,ab,kw | **177,422** |
| **#2** | 'burn'/de OR 'chemical burn'/exp OR 'burn':ti,ab,kw OR 'burns':ti,ab,kw OR 'scald*':ti,ab,kw OR 'postburn*':ti,ab,kw OR ('thermal':ti,ab,kw AND 'injur*':ti,ab,kw) | **129,418** |
| **#1** | adolescen*:ti,ab,kw OR 'adolescence'/exp OR 'adolescent coping orientation for problem experiences'/exp OR 'adolescent development'/exp OR 'adolescent disease'/exp OR 'adolescent health'/exp OR 'adolescent parent'/exp OR 'adolescent pregnancy'/exp OR 'adolescent smoking'/exp OR 'adolescent'/exp OR 'adolescent-family inventory of life events and changes'/exp OR babies:ti,ab,kw OR baby:ti,ab,kw OR 'birth weight'/exp OR boy:ti,ab,kw OR boyhood:ti,ab,kw OR boys:ti,ab,kw OR 'brazelton neonatal behavioral assessment scale'/exp OR 'child abuse'/exp OR 'child advocacy'/exp OR 'child behavior checklist'/exp OR 'child behavior'/exp OR 'child care'/exp OR 'child death'/exp OR 'child health care'/exp OR 'child health'/exp OR 'child nutrition'/exp OR 'child parent relation'/exp OR 'child psychology'/exp OR 'child restraint system'/exp OR 'child safety'/exp OR 'child welfare'/exp OR child*:ti,ab,kw OR 'child'/exp OR 'childhood disease'/exp OR 'childhood mortality'/exp OR 'childhood'/exp OR girl:ti,ab,kw OR girlhood:ti,ab,kw OR girls:ti,ab,kw OR 'high risk infant'/exp OR infan*:ti,ab,kw OR 'infant disease'/exp OR 'infant mortality'/exp OR 'infant nutrition'/exp OR 'infant welfare'/exp OR 'infanticide'/exp OR 'infantile diarrhea'/exp OR 'infantile hypotonia'/exp OR 'juvenile delinquency'/exp OR neonat*:ti,ab,kw OR 'neonatal weight loss'/exp OR 'newborn disease'/exp OR 'newborn morbidity'/exp OR 'newborn period'/exp OR newborn*:ti,ab,kw OR 'newborn'/exp OR nicu:ti,ab,kw OR 'only child'/exp OR paediatr*:ti,ab,kw OR pediatr*:de,ab,ti,kw OR 'pediatric advanced life support'/exp OR 'pediatric anesthesia'/exp OR 'pediatric cardiology'/exp OR 'pediatric hospital'/exp OR 'pediatric intensive care nursing'/exp OR 'pediatric nurse practitioner'/exp OR 'pediatric nursing'/exp OR 'pediatric rehabilitation'/exp OR 'pediatric surgery'/exp OR 'newborn hypoxia'/exp OR 'pediatric ward'/exp OR 'pediatrics'/exp OR perinat*:ti,ab,kw OR 'perinatal development'/exp OR 'perinatal period'/exp OR 'persistent hyperinsulinemic hypoglycemia of infancy'/exp OR picu:ti,ab,kw OR postnat*:ti,ab,kw OR 'postnatal care'/exp OR 'postnatal development'/exp OR 'postnatal growth'/exp OR postneonat*:ti,ab,kw OR preschool*:ti,ab,kw OR puberty:ti,ab,kw OR 'runaway behavior'/exp OR 'school child':ti,ab,kw OR schoolchild*:ti,ab,kw OR 'severe myoclonic epilepsy in infancy'/exp OR suckling*:ti,ab,kw OR teen:ti,ab,kw OR teenager*:ti,ab,kw OR teens:ti,ab,kw OR toddler*:ti,ab,kw OR 'transient hypogammaglobulinemia of infancy'/exp OR youth:ti,ab,kw OR youths:ti,ab,kw | **6,898,978** |

**Search strategy for Clarivate Analytics/Web of Science Core Collection (26 March 2024)**

| **Search** | **Query** | **Results** |
| --- | --- | --- |
| **#4** | #1 AND #2 AND #3 | **891** |
| **#3** | TS=("cicatri*" OR "scar" OR "scars" OR "scarring" OR "keloid*") | **101,483** |
| **#2** | TS=(("chemical" AND "Burn*") OR "burn" OR "burns" OR "scald*" OR "postburn*" OR ("thermal" AND "injur*")) | **125,229** |
| **#1** | TS=("infan*" OR "child*" OR "adolescen*" OR "pediatric*" OR "paediatric*" OR "pube*" OR "juvenil*" OR "school*" OR "newborn*" OR "new-born*" OR "neo-nat*" OR "neonat*" OR "premature*" OR "postmature*" OR "pre-mature*" OR "post-mature*" OR "preterm*" OR "pre-term*" OR "baby" OR "babies" OR "toddler*" OR "youngster*" OR "preschool*" OR "kindergart*" OR "kid" OR "kids" OR "playgroup*" OR "play-group*" OR "playschool*" OR "prepube*" OR "preadolescen*" OR "junior high*" OR "highschool*" OR "senior high" OR "young people*" OR "minors") | **4,468,985** |
