# Supplementary material for: How to Assess Scar Quality in Pediatric Burn Patients: A Systematic Review on the Type and Content of Outcome Measurement Instruments
Source: J Burn Care Res. 2025 May 3;46(6):1294–301. doi: 10.1093/jbcr/iraf048 (PMC12596693; doi:10.1093/jbcr/iraf048)
Supplement: iraf048_suppl_Supplementary_Material [file iraf048_suppl_supplementary_material.docx]

Supplementary Digital Content 2a. Overview of **clinician-reported** outcome measures, items included and frequency

|  | Nr. of studies | Nr. of items used | Vascularity | Pigmentation | Color | Thickness | Relief | Texture / roughness | Pliability | Elasticity | Tension | Hardness / consistency | Hydration | Surface area / irregularities | Distortion | Contour | Sensation (to touch) | Hair growth | Size | Shiny | Volume | Distensibility | Scar pockets | Itch | Pain | Subjective score / improvement / overall opinion / improvement | Severity of the lesions | Presence of defects / Inflammation / Maceration |
| --- | --- | --- | --- | --- | --- | --- | --- | --- | --- | --- | --- | --- | --- | --- | --- | --- | --- | --- | --- | --- | --- | --- | --- | --- | --- | --- | --- | --- |
| VSS [Sullivan] ^1–54^ | 54 | 4 | 54 | 54 |  | 54 |  |  | 54 |  |  |  |  |  |  |  |  |  |  |  |  |  |  |  |  |  |  |  |
| 1 item use ^55^ | 1 | 1 |  |  |  | 1 |  |  |  |  |  |  |  |  |  |  |  |  |  |  |  |  |  |  |  |  |  |  |
| 2 item use ^56^ | 1 | 2 | 1 | 1 |  |  |  |  |  |  |  |  |  |  |  |  |  |  |  |  |  |  |  |  |  |  |  |  |
| 3 item use ^57,58^ | 2 | 3 | 2 |  |  | 2 |  |  | 2 |  |  |  |  |  |  |  |  |  |  |  |  |  |  |  |  |  |  |  |
| Modified ^59^ | 1 | 4 | 1 | 1 |  | 1 |  |  | 1 |  | ` |  |  |  |  |  |  |  |  |  |  |  |  |  |  |  |  |  |
| Modified ^60^ | 1 | 3 | 1 |  |  | 1 |  |  | 1 |  |  |  |  |  |  |  |  |  |  |  |  |  |  |  |  |  |  |  |
| Modified ^61^ | 1 | 5 | 1 | 1 |  | 1 |  |  | 1 |  |  |  |  |  |  |  |  |  | 1 |  |  |  |  |  |  |  |  |  |
| Modified ^62^ | 1 | 4 | 1 | 1 |  | 1 |  |  | 1 |  |  |  |  |  |  |  |  |  |  |  |  |  |  |  |  |  |  |  |
| Modified ^63^ | 1 | 6 | 1 | 1 |  | 1 |  |  | 1 |  |  |  |  |  |  |  |  |  |  |  |  |  |  | 1 |  | 1 |  |  |
| mVSS [Baryza] ^64–95^ | 32 | 4 | 32 | 32 |  | 32 |  |  | 32 |  |  |  |  |  |  |  |  |  |  |  |  |  |  |  |  |  |  |  |
| 1 item use ^96^ | 1 | 1 |  | 1 |  |  |  |  |  |  |  |  |  |  |  |  |  |  |  |  |  |  |  |  |  |  |  |  |
| 1 item use ^97,98^ | 2 | 1 |  |  |  | 2 |  |  |  |  |  |  |  |  |  |  |  |  |  |  |  |  |  |  |  |  |  |  |
| 2 item use ^99,100^ | 2 | 2 |  |  |  | 2 |  |  | 2 |  |  |  |  |  |  |  |  |  |  |  |  |  |  |  |  |  |  |  |
| 3 item use ^101^ | 1 | 3 | 1 |  |  | 1 |  |  | 1 |  |  |  |  |  |  |  |  |  |  |  |  |  |  |  |  |  |  |  |
| Modified ^102^ | 1 | 4 | 1 | 1 |  | 1 |  |  | 1 |  |  |  |  |  |  |  |  |  |  |  |  |  |  |  |  |  |  |  |
| Modified ^103,104^ | 2 | 5-6 | 2 | 1 |  | 2 |  |  | 1 |  |  | 1 |  |  |  |  |  |  |  |  |  |  |  | 2 | 1 |  |  | 1 |
| VSS [Nedelec] ^105–108^ | 4 | 6 | 4 | 4 |  | 4 |  |  | 4 |  |  |  |  |  |  |  |  |  |  |  |  |  |  | 4 | 4 |  |  |  |
| 5 item use ^109^ | 1 | 5 | 1 | 1 |  |  |  |  | 1 |  |  |  |  |  |  |  |  |  |  |  |  |  |  | 1 | 1 |  |  |  |
| Modified ^110–112^ | 4 | 4 | 3 | 3 |  | 3 |  |  | 3 |  |  |  |  |  |  |  |  |  |  |  |  |  |  |  |  |  |  |  |
| Modified ^113–119^ | 7 | 4 | 7 | 7 |  | 7 |  |  | 7 |  |  |  |  |  |  |  |  |  |  |  |  |  |  |  |  |  |  |  |
| Modified ^120^ | 1 | 5 | 1 |  |  | 1 |  |  |  |  |  | 1 |  |  |  |  |  |  |  |  |  |  |  | 1 |  |  |  | 1 |
| Modified ^121^ | 1 | 3 | 1 | 1 |  | 1 |  |  |  |  |  |  |  |  |  |  |  |  |  |  |  |  |  |  |  |  |  |  |
|  | Nr. of studies | Nr. of items used | Vascularity | Pigmentation | Color | Thickness | Relief | Texture / roughness | Pliability | Elasticity | Tension | Hardness / consistency | Hydration | Surface area / irregularities | Distortion | Contour | Sensation (to touch) | Hair growth | Size | Shiny | Volume | Distensibility | Scar pockets | Itch | Pain | Subjective score / improvement / overall opinion / improvement | Severity of the lesions | Presence of defects / Inflammation / Maceration |
| Modified ^122^ | 1 | 4 | 1 | 1 |  | 1 |  |  | 1 |  |  |  |  |  |  |  |  |  |  |  |  |  |  |  |  |  |  |  |
| Modified ^123^ | 1 | 4 | 1 | 1 |  | 1 |  |  | 1 |  |  |  |  |  |  |  |  |  |  |  |  |  |  |  |  |  |  |  |
| Modified ^123^ | 1 | 4 | 1 | 1 |  | 1 |  |  | 1 |  |  |  |  |  |  |  |  |  |  |  |  |  |  |  |  |  |  |  |
| Modified ^124,125^ | 2 | 4 | 2 | 2 |  | 2 |  |  | 2 |  |  |  |  |  |  |  |  |  |  |  |  |  |  |  |  |  |  |  |
| Unspecified version of VSS ^121,126–143^ | 19 | 2-9 | 19 | 18 | 1 | 19 |  | 1 | 16 |  |  | 1 |  |  |  |  | 1 | 1 |  |  |  |  |  | 2 | 1 | 1 |  | 1 |
| Unspecified version and items of VSS ^108,144–163^ | 21 |  |  |  |  |  |  |  |  |  |  |  |  |  |  |  |  |  |  |  |  |  |  |  |  |  |  |  |
| Vancouver General Hospital Scar index ^164^ | 1 | 4 | 1 |  | 1 | 1 |  |  | 1 |  |  |  |  |  |  |  |  |  |  |  |  |  |  |  |  |  |  |  |
| Vancouver Burn Skin Score ^165^ | 1 | 4 | 1 | 1 |  |  |  |  | 1 | 1 |  |  |  |  |  |  |  |  |  |  |  |  |  |  |  |  |  |  |
| Vancouver Burn Scar Assessment Scale ^166,167^ | 2 | 6 | 2 | 2 |  | 2 |  |  | 2 |  |  |  |  |  |  |  |  |  |  |  |  |  |  | 2 | 2 |  |  |  |
| Scar Regression Score ^168^ | 1 | 3 | 1 | 1 |  | 1 |  |  |  |  |  |  |  |  |  |  |  |  |  |  |  |  |  |  |  |  |  |  |
| Modified Inventory of Potential Reconstructive Needs ^169,170^ | 2 | 4 |  | 2 |  | 2 |  |  | 2 |  |  |  |  |  |  |  |  |  |  |  |  |  | 2 |  |  |  |  |  |
| POSAS, observer scale 1.0 ^1,24,41,68,72,81,91,159,171–178^ | 16 | 5 | 16 | 16 |  | 16 | 16 |  | 16 |  |  |  |  |  |  |  |  |  |  |  |  |  |  |  |  |  |  |  |
| 1 item use ^179^ | 1 | 1 |  |  |  |  |  |  | 1 |  |  |  |  |  |  |  |  |  |  |  |  |  |  |  |  |  |  |  |
| 2 item use ^96^ | 1 | 2 | 1 | 1 |  |  |  |  |  |  |  |  |  |  |  |  |  |  |  |  |  |  |  |  |  |  |  |  |
| 4 item use ^180^ | 1 | 4 |  | 1 |  | 1 | 1 |  | 1 |  |  |  |  |  |  |  |  |  |  |  |  |  |  |  |  |  |  |  |
| Modified ^181^ | 1 | 5 | 1 | 1 |  | 1 |  |  |  | 1 | 1 |  |  |  |  |  |  |  |  |  |  |  |  |  |  |  |  |  |
| POSAS, observer scale 2.0 ^6,7,16,17,23,70,75,122,129,160,182–195^ | 24 | 7 | 24 | 24 |  | 24 | 24 |  | 24 |  |  |  |  | 24 |  |  |  |  |  |  |  |  |  |  |  | 24 |  |  |
|  | Nr. of studies | Nr. of items used | Vascularity | Pigmentation | Color | Thickness | Relief | Texture / roughness | Pliability | Elasticity | Tension | Hardness / consistency | Hydration | Surface area / irregularities | Distortion | Contour | Sensation (to touch) | Hair growth | Size | Shiny | Volume | Distensibility | Scar pockets | Itch | Pain | Subjective score / improvement / overall opinion / improvement | Severity of the lesions | Presence of defects / Inflammation / Maceration |
| 2 item use ^196,197^ | 2 | 2 | 1 | 2 |  |  |  |  |  |  |  |  |  |  |  |  |  |  |  |  |  |  |  |  |  | 1 |  |  |
| 5 item use ^77^ | 1 | 5 | 1 | 1 |  | 1 |  |  | 1 |  |  |  |  |  |  |  |  |  |  |  |  |  |  |  |  | 1 |  |  |
| 6 item use ^198–200^ | 3 | 6 | 3 | 3 |  | 3 | 2 | 1 | 3 |  |  |  |  | 3 |  |  |  |  |  |  |  |  |  |  |  |  |  |  |
| Modified POSAS observer scale, unspecified version ^201,202^ | 2 | 2-5 | 2 | 2 |  | 1 |  |  | 1 |  |  |  |  | 1 |  |  |  |  |  |  |  |  |  |  |  |  |  |  |
| Modified POSAS observer scale, unspecified version and items ^158,203^ | 2 |  |  |  |  |  |  |  |  |  |  |  |  |  |  |  |  |  |  |  |  |  |  |  |  |  |  |  |
| Modified POSAS observer scale, unspecified version ^204^ | 1 | 7 |  |  |  | 1 | 1 |  | 1 |  |  |  |  | 1 |  |  |  |  |  |  |  |  |  | 1 | 1 | 1 |  |  |
| Seattle Scar Scale ^52,94,205^ | 3 | 4 |  | 3 |  | 6 |  | 3 |  |  |  |  |  |  |  |  |  |  |  |  |  |  |  |  |  |  |  |  |
| Modified ^206^ | 1 | 4 |  | 1 |  | 1 |  | 1 |  |  |  |  |  |  |  |  |  |  |  |  |  |  |  |  |  |  |  |  |
| Toronto Pediatric Itch Scale ^78,207^ | 2 | 2 |  |  |  |  |  |  |  |  |  |  |  |  |  |  |  |  |  |  |  |  |  | 2 |  |  |  |  |
| Hamilton Scar Scale ^52,94,208–210^ | 5 | 4 | 5 | 5 |  | 5 |  |  |  |  |  |  |  | 5 |  |  |  |  |  |  |  |  |  |  |  |  |  |  |
| Manchester Scar Score ^122,211,212^ | 3 | 5 |  |  | 3 |  |  | 3 |  |  |  |  |  |  | 3 | 3 |  |  |  | 3 |  |  |  |  |  |  |  |  |
| Modified ^213,214^ | 2 | 3 |  |  | 2 |  |  | 2 |  |  |  |  |  |  | 2 |  |  |  |  |  |  |  |  |  |  |  |  |  |
| Modified ^215^ | 1 | 4 |  |  | 1 |  |  | 1 |  |  |  |  |  |  |  | 1 | 1 |  |  |  |  |  |  |  |  |  |  |  |
| Kyoto scar scale ^216^ | 1 | 2 | 1 |  |  |  |  |  |  |  |  | 1 |  |  |  |  |  |  |  |  |  |  |  |  |  |  |  |  |
| Scar Assessment Scale by the Plastic Surgery Department of Sao Paulo University Medical School Hospital ^217^ | 1 | 5 |  |  | 1 |  |  |  |  |  |  |  | 1 | 1 |  |  |  |  |  |  | 1 | 1 |  |  |  |  |  |  |
|  | Nr. of studies | Nr. of items used | Vascularity | Pigmentation | Color | Thickness | Relief | Texture / roughness | Pliability | Elasticity | Tension | Hardness / consistency | Hydration | Surface area / irregularities | Distortion | Contour | Sensation (to touch) | Hair growth | Size | Shiny | Volume | Distensibility | Scar pockets | Itch | Pain | Subjective score / improvement / overall opinion / improvement | Severity of the lesions | Presence of defects / Inflammation / Maceration |
| **Subtotal** | 247 | 1-9 | 200 | 199 | 9 | 209 | 44 | 12 | 188 | 2 | 1 | 4 | 1 | 35 | 5 | 4 | 2 | 1 | 1 | 3 | 1 | 1 | 2 | 16 | 10 | 29 |  | 3 |
| **Unlabeled** ^6,16,20,51,62,142,213,214,218–269^ | 61 | 1-5 | 11 | 13 | 21 | 24 | 1 | 10 | 6 | 1 |  | 8 | 1 | 4 | 2 | 1 |  |  | 1 |  |  | 1 |  | 3 | 2 | 18 | 1 | 1 |
| **Total** | 308 | 1-9 | **211** | **212** | 30 | **233** | 45 | 22 | 194 | 3 | 1 | 12 | 2 | 39 | 7 | 5 | 2 | 1 | 2 | 3 | 1 | 2 | 2 | 19 | 12 | 47 | 1 | 4 |

Supplementary Digital Content 2b. Overview of **patient-reported** outcome measures, items included and frequency

|  | Nr. of studies | Nr. of items used | Vascularity | Pigmentation | Color | Thickness | Relief / wrinkled / lumpy | Irregularity | Texture / Roughness | Pliability / Stiffness | Hardness | Surface area / distortion | Contour | Width / Length / Size | Tight / pulling skin | Pain | Itch | Hydration | Loss of sensation / numbness | Paresthesia / abnormal sensation / uncomfortable sensations | Distensibility | Volume | (Hyper)sensitivity | Burning sensation | Dry skin | Fragile skin | Sensitive for temperature (changes) | Open wounds | Sense of tension | Subjective score | Tenderness upon palpation | Swelling |
| --- | --- | --- | --- | --- | --- | --- | --- | --- | --- | --- | --- | --- | --- | --- | --- | --- | --- | --- | --- | --- | --- | --- | --- | --- | --- | --- | --- | --- | --- | --- | --- | --- |
| Scar Assessment Scale by the Plastic Surgery Department of Sao Paulo University Medical School Hospital ^217^ | 1 | 6 |  |  | 1 |  |  |  |  |  |  | 1 |  |  |  |  |  | 1 |  |  | 1 | 1 |  |  |  |  |  |  |  | 1 |  |  |
| 5-D Pruritus Scale Questionnaire ^18,19^ | 2 | 1 |  |  |  |  |  |  |  |  |  |  |  |  |  |  | 2 |  |  |  |  |  |  |  |  |  |  |  |  |  |  |  |
| Itch Man Scale ^17,55,57^ | 3 | 1 |  |  |  |  |  |  |  |  |  |  |  |  |  |  | 3 |  |  |  |  |  |  |  |  |  |  |  |  |  |  |  |
| Wrong-Baker Faces scale ^57^ | 1 | 1 |  |  |  |  |  |  |  |  |  |  |  |  |  | 1 |  |  |  |  |  |  |  |  |  |  |  |  |  |  |  |  |
| UNC-4P ^14,110,112,270^ | 4 | 4 |  |  |  |  |  |  |  | 4 |  |  |  |  |  | 4 | 4 |  |  | 4 |  |  |  |  |  |  |  |  |  |  |  |  |
| Modified Burn Injury Questionnaire by Fisher ^271^ | 1 | 2 |  |  |  |  |  |  |  |  |  |  |  |  |  |  |  |  | 1 | 1 |  |  |  |  |  |  |  |  |  |  |  |  |
| Visual Analogue Scale ^9,18,71,79,90,102,106,118,119,151,172,212^ | 11 | 1-5 |  |  | 2 | 1 |  |  |  | 1 | 1 |  |  |  | 1 | 7 | 8 |  |  | 1 |  |  |  |  | 2 |  |  |  |  | 3 |  |  |
| Numeric Rating Scale ^55^ | 1 | 1 |  |  |  |  |  |  |  |  |  |  |  |  |  |  | 1 |  |  |  |  |  |  |  |  |  |  |  |  |  |  |  |
| Michigan Hand Questionnaire ^85,202,272^ | 3 | 3 |  |  |  |  |  |  |  |  |  |  |  |  |  | 3 |  |  |  |  |  |  |  |  |  |  |  |  |  | 3 |  |  |
| BBSIP, feelings ^173,188,189,273–275^ | 6 | 3 |  |  |  |  |  |  |  |  |  |  |  |  |  | 6 | 6 |  |  | 6 |  |  |  |  |  |  |  |  |  |  |  |  |
| BBSIP, what scars are like ^173,188,189,273–275^ | 6 | 8 |  |  | 6 | 6 | 6 |  | 6 |  | 6 |  |  |  | 6 |  |  |  |  |  |  |  |  |  | 6 |  |  | 6 |  |  |  |  |
| Scar-Q, appearance scale ^195,276^ | 2 | 7 |  |  | 4 | 2 | 2 |  |  |  |  |  | 2 | 4 |  |  |  |  |  |  |  |  |  |  |  |  |  |  |  |  |  |  |
| Scar-Q, symptom scale ^195,276^ | 2 | 11 |  |  |  |  |  |  |  |  | 2 |  |  |  | 4 | 2 | 2 |  | 2 | 2 |  |  | 2 |  | 2 |  | 2 |  |  |  |  | 2 |
| mVSS [Baryza] ^76,277^ | 2 | 4 | 2 | 2 |  | 2 |  |  |  | 2 |  |  |  |  |  |  |  |  |  |  |  |  |  |  |  |  |  |  |  |  |  |  |
| mVSS ^124^ | 1 | 4 | 1 | 1 |  | 1 |  |  |  | 1 |  |  |  |  |  |  |  |  |  |  |  |  |  |  |  |  |  |  |  |  |  |  |
| Kyoto scar scale ^216^ | 1 | 2 |  |  |  |  |  |  |  |  |  |  |  |  |  | 1 | 1 |  |  |  |  |  |  |  |  |  |  |  |  |  |  |  |
| Scar Problems Questionnaire ^278^ | 1 | 10 |  |  | 1 | 1 |  |  |  |  |  |  |  |  | 1 | 1 | 1 |  | 1 |  |  |  | 1 |  | 1 | 1 |  | 1 |  |  |  |  |
| Itch Severity Scale ^279^ | 1 | 1 |  |  |  |  |  |  |  |  |  |  |  |  |  |  | 1 |  |  |  |  |  |  |  |  |  |  |  |  |  |  |  |
| Toronto Pediatric Itch Scale ^173^ | 1 | 1 |  |  |  |  |  |  |  |  |  |  |  |  |  |  | 1 |  |  |  |  |  |  |  |  |  |  |  |  |  |  |  |
| POSAS patient scale 1.0 ^1,41,54,72,81,172–177,181,186,187,196,201,273,280–282^ | 20 | 6 |  |  | 20 | 20 |  | 20 |  | 20 |  |  |  |  |  | 20 | 20 |  |  |  |  |  |  |  |  |  |  |  |  |  |  |  |
| 1 item use ^173^ | 1 | 1 |  |  |  |  |  |  |  |  |  |  |  |  |  |  | 1 |  |  |  |  |  |  |  |  |  |  |  |  |  |  |  |
| POSAS patient scale 2.0 ^7,16,23,70,75,77,129,180,182–185,188–194,199,203,283–289^ | 30 | 7 |  |  | 30 | 30 |  | 30 |  | 30 |  |  |  |  |  | 30 | 30 |  |  |  |  |  |  |  |  |  |  |  |  | 30 |  |  |
| Modified ^178^ | 1 | 5 |  |  | 1 |  |  | 1 |  | 1 |  |  |  |  |  | 1 | 1 |  |  |  |  |  |  |  |  |  |  |  |  |  |  |  |
| POSAS, unknown version and items ^31,142,290^ | 3 |  |  |  |  |  |  |  |  |  |  |  |  |  |  |  |  |  |  |  |  |  |  |  |  |  |  |  |  |  |  |  |
| **Subtotal** | 105 | 1-11 | 3 | 3 | 65 | 63 | 8 | 51 | 6 | 59 | 9 | 1 | 2 | 4 | 11 | 77 | 82 | 1 | 4 | 14 | 1 | 1 | 3 | 0 | 11 | 1 | 2 | 7 | 0 | 37 | 0 | 2 |
| Unlabeled ^57,79,86,111,129,172,215,219,221,226,227,229,231,249,252,253,262,271,291–295^ | 25 | 1-6 | 3 | 3 | 2 | 4 | 1 | 0 | 3 | 2 | 2 | 0 | 1 | 1 | 0 | 8 | 13 | 0 | 1 | 3 | 0 | 0 | 1 | 2 | 1 | 1 | 1 | 0 | 1 | 9 | 1 | 0 |
| **Total** | 129 | 1-11 | 6 | 6 | **67** | **67** | 9 | 51 | 9 | 61 | 11 | 1 | 3 | 5 | 11 | **84** | **95** | 1 | 5 | 17 | 1 | 1 | 4 | 2 | 12 | 2 | 3 | 6 | 1 | 46 | 1 | 2 |

Supplementary Digital Content 2c. Any specific (age-related) conditions for filling out the PROM.

| **Type of PROM** | **Condition for filling out** | **Number of studies  reporting this condition** |
| --- | --- | --- |
| **POSAS 1.0** |  |  |
|  | With support of parent ^184,283^ | 2 |
|  | With support of parent if <12 y/o ^172^ | 1 |
|  | Children from 5 y/o ^173,175,280^ | 3 |
|  | Children from 8 y/o ^185,273^ | 2 |
|  | Children from 10 y/o ^190^ | 1 |
|  | Children from 12 y/o ^284,294^ | 2 |
|  | Children from 16 y/o ^270^ | 1 |
|  | Patient or parent ^186^ | 1 |
|  | Parent or caregiver ^173,181^  *Reported in* | 2 |
|  |  | *15/21 (71%) of studies using POSAS 1.*0 |
| **POSAS 2.0** |  |  |
|  | Children from 6 y/o ^192^ | 1 |
|  | Children from 8 y/o ^185^ | 1 |
|  | Children from 10 y/o ^190^ | 1 |
|  | Children from 12 y/o ^284^ | 1 |
|  | Children from 13 y/o ^287^ | 1 |
|  | Patient or parent ^129,194,285^ | 3 |
|  | With support of parent if needed ^23,77,182,184,283^ | 5 |
|  | Parent or caregiver ^75,189,191,289^  *Reported in* | 4 |
|  |  | *17/31 (55%) of studies using POSAS 2.0* |
| **Michigan Hand Questionnaire** | | |
|  | Parents ^85^  *Reported in* | 1 |
|  |  | *1/3 (33%) of studies using MHQ* |
| **BBSIP** | | |
|  | Children from 8 y/o ^173,273^ | 2 |
|  | Parent or caregiver ^189^  *Reported in* | 1 |
|  |  | *3/6 (50%) of studies using BBSIP* |
| **(m)VSS** | | |
|  | Parents ^76,124^  *Reported in* | 2 |
|  |  | *2/3 (75%) of studies using a (m)VSS* |
| **Modified Burn Injury Questionnaire** | | |
|  | Parents ^271^ | 1 |
|  | *Reported in* | *1/1 (100%) of studies using modified Burn Injury  Questionnaire* |
| **Itch Man Scale** | | |
|  | Only the caregiver (for children <6 y/o) or both the caregiver and child ^55^ | 1 |
|  | *Reported in* | *1/3 (33%) of studies using Itch Man Scale* |
| **Itch Severity Scale** | | |
|  | Parents ^279^ | 1 |
|  | *Reported in* | *1/1 (100%) of studies using Itch Severity Scale* |
| **Toronto Pediatric Itch Scale** | | |
|  | Children from 5 y/o ^173^ | 1 |
|  | *Reported in* | *1/1 (100%) of studies using Toronto Pediatric Itch Scale* |
| **Visual Analogue Scale** | | |
|  | Patient or parent ^151^ | 1 |
|  | Parents in the case of young children ^9^ | 1 |
|  | Parents ^151,212^ | 2 |
|  | *Reported in* |  |
| **Scar Problems Questionnaire** | | |
|  | With support of parent ^278^ | 1 |
|  | *Reported in* | *1/1 (100%) of studies using Scar Problems Questionnaire* |
| **Numeric Rating Scale, itch** | | |
|  | Only the caregiver or both the caregiver and child ^55^ | 1 |
|  | *Reported in* |  |
| **UNC-4P** | | |
|  | Children from 16 y/o ^270^ | 1 |
|  | *Reported in* | *1/4 (25%) of studies using UNC-4P* |
| **Unlabeled** | | |
|  | Parents ^57,271^ | 2 |
|  | *Reported in* |  |
| **Total number of studies reporting (age-related) conditions** | | **52/129 (42%) of studies using a PROM** |

BBSIP = Brisbane Burn Scar Impact Profile, POSAS = Patient and Observer Scar Assessment Scale, MHQ = Michigan Hand Questionnaire, UNC-4P = University of North Carolina ‘’4P’’ Scar Scale, (m)VSS = (modified) Vancouver Scar Scale

Supplementary Digital Content 2d. Overview of **measurement** **devices**, included items and frequency of occurrence

|  | | Nr. of studies | Nr. of constructs measured | Thickness | Density | Stiffness | Extensibility | Erythema | Color | Pigmentation | Surface area | Transepidermal water loss | Sebum excretion | Size | Hardness | Volume | Hydration | Oxygen tension | Scar surface temperature |
| --- | --- | --- | --- | --- | --- | --- | --- | --- | --- | --- | --- | --- | --- | --- | --- | --- | --- | --- | --- |
| Ultrasound ^76,77,79,82,91,102,115,116,121,153,163,164,167,172,173,185,193,201,204,226,259,273,277,292,296–300^ | | 29 | 1-2 | 29 | 1 | 1 |  |  |  |  |  |  |  |  |  |  |  |  |  |
| Acoustic Radiation Force Impulse ultrasound elastography ^77^ | | 1 | 1 |  |  | 1 |  |  |  |  |  |  |  |  |  |  |  |  |  |
| Tissue Ultrasound Palpation System ^8^ | | 1 | 1 | 1 |  |  |  |  |  |  |  |  |  |  |  |  |  |  |  |
| Colour power doppler echography ^79,301^ | | 2 | 1 |  |  |  |  | 2 |  |  |  |  |  |  |  |  |  |  |  |
| Color meters | |  |  |  |  |  |  |  |  |  |  |  |  |  |  |  |  |  |  |
| Computer analysis of photographs ^4,32,180^ | | 3 | 1 |  |  |  |  |  | 2 | 1 |  |  |  |  |  |  |  |  |  |
| Dermascopy ^56^ | | 1 | 2 |  |  |  |  | 1 |  | 1 |  |  |  |  |  |  |  |  |  |
| Narrow-band spectrophotometric color analysis | |  |  |  |  |  |  |  |  |  |  |  |  |  |  |  |  |  |  |
|  | Dermaspectrometer ^13,63,94,96,142,173,175,197,201,228,249,273^ | 12 | 1-2 |  |  |  |  | 11 |  | 8 |  |  |  |  |  |  |  |  |  |
|  | Mexameter ^172,196,197,299^ | 4 | 2 |  |  |  |  | 1 |  | 1 |  |  |  |  |  |  |  |  |  |
|  | MiniScan XE Plus spectrocolorimeter ^56^ | 1 | 1 |  |  |  |  | 1 |  | 1 |  |  |  |  |  |  |  |  |  |
| Tristimulus color systems | |  |  |  |  |  |  |  |  |  |  |  |  |  |  |  |  |  |  |
|  | Minolta Chromameter ^94,96,292,296,302^ | 5 | 1 |  |  |  |  |  | 5 |  |  |  |  |  |  |  |  |  |  |
|  | Labscan XE ^303^ | 1 | 1 |  |  |  |  |  | 1 |  |  |  |  |  |  |  |  |  |  |
|  | Colorimeter ^23,80,197^ | 3 | 1-2 |  |  |  |  | 1 | 2 | 1 |  |  |  |  |  |  |  |  |  |
| Biomechanical properties | |  |  |  |  |  |  |  |  |  |  |  |  |  |  |  |  |  |  |
| Cutometer ^16,23,37,63,70,153,179,182,255,259,283,292,299,304,305^ | | 16 | 1 |  |  | 16 |  |  |  |  |  |  |  |  |  |  |  |  |  |
| Durometer ^94,187,193,296^ | | 4 | 1 |  |  |  |  |  |  |  |  |  |  |  | 4 |  |  |  |  |
| Extensometer ^306^ | | 1 | 1 |  |  |  | 1 |  |  |  |  |  |  |  |  |  |  |  |  |
| Dermaflex A ^249^ | | 1 | 1 |  |  | 1 |  |  |  |  |  |  |  |  |  |  |  |  |  |
| Dermal Torque Meter ^201,240^ | | 2 | 1 |  |  | 2 |  |  |  |  |  |  |  |  |  |  |  |  |  |
| Biomedical Tissue Characterization system ^4^ | | 1 | 1 |  |  | 1 |  |  |  |  |  |  |  |  |  |  |  |  |  |
| Pneumatonometer ^94^ | | 1 | 1 |  |  | 1 |  |  |  |  |  |  |  |  |  |  |  |  |  |
| ASTM D882-67 test ^306^ | | 1 | 1 |  |  |  | 1 |  |  |  |  |  |  |  |  |  |  |  |  |
| Modified ASTM D882-67 test ^258^ | | 1 | 1 |  |  |  | 1 |  |  |  |  |  |  |  |  |  |  |  |  |
| Nimble ^182,283^ | | 2 | 1 |  |  | 1 |  |  |  |  |  |  |  |  |  |  |  |  |  |
| Scar dimensions | |  |  |  |  |  |  |  |  |  |  |  |  |  |  |  |  |  |  |
| Caliper/ruler ^13,138,139,219,228,236,307–309^ | | 9 | 1-2 | 8 |  |  |  |  |  |  |  |  |  | 4 |  |  |  |  |  |
| Planimetry ^37,63,82,310^ | | 4 | 1 | 1 |  |  |  |  |  |  | 1 |  |  | 1 |  |  |  |  |  |
| Mold/negative impressions^9,106,311–314^ | | 6 | 1-2 | 1 |  |  |  |  |  |  | 1 |  |  |  |  | 5 |  |  |  |
| 3D imaging ^4,138,139,185,204,315–317^ | | 8 | 1-3 | 4 |  |  |  | 2 |  | 2 | 3 |  |  |  |  | 1 |  |  |  |
| Dermascan A ^309^ | | 1 | 1 | 1 |  |  |  |  |  |  |  |  |  |  |  |  |  |  |  |
| Dermascan C ^249^ | | 1 | 3 | 1 | 1 |  |  |  |  |  | 1 |  |  |  |  |  |  |  |  |
| Image Tool® ^52,94^ | | 2 | 1 | 2 |  |  |  |  |  |  |  |  |  |  |  |  |  |  |  |
| Moisture meters | |  |  |  |  |  |  |  |  |  |  |  |  |  |  |  |  |  |  |
| ASA M-2 ^64,80^ | | 2 | 1-2 | 1 |  |  |  |  |  |  |  | 2 |  |  |  |  |  |  |  |
| Tewameter ^172,299^ | | 2 | 1 |  |  |  |  |  |  |  |  | 1 |  |  |  |  |  |  |  |
| ASTM E96-66 method ^306^ | | 1 | 1 |  |  |  |  |  |  |  |  | 1 |  |  |  |  |  |  |  |
| Sebumeter ^172^ | | 1 | 1 |  |  |  |  |  |  |  |  |  | 1 |  |  |  |  |  |  |
| Laser imaging | |  |  |  |  |  |  |  |  |  |  |  |  |  |  |  |  |  |  |
| Laser Doppler Perfusion Imaging ^121,163^ | | 2 | 1 |  |  |  |  | 2 |  |  |  |  |  |  |  |  |  |  |  |
| Laser Doppler Perfusion Monitor ^318^ | | 1 | 1 |  |  |  |  | 1 |  |  |  |  |  |  |  |  |  |  |  |
| Laser Doppler Flowmeter ^94,218,319^ | | 3 | 1 |  |  |  |  | 3 |  |  |  |  |  |  |  |  |  |  |  |
| Oxygenation measurement | |  |  |  |  |  |  |  |  |  |  |  |  |  |  |  |  |  |  |
| Oximeter ^226^ | | 1 | 1 |  |  |  |  |  |  |  |  |  |  |  |  |  |  | 1 |  |
| Radiometer TCM1 Transcutaneous Oxygen Monitor ^306^ | | 1 | 1 |  |  |  |  |  |  |  |  |  |  |  |  |  |  | 1 |  |
| Topography | |  |  |  |  |  |  |  |  |  |  |  |  |  |  |  |  |  |  |
| Infra-red camera ^226^ | | 1 | 1 |  |  |  |  |  |  |  |  |  |  |  |  |  |  |  | 1 |
| Digital thermometer ^306^ | | 1 | 1 |  |  |  |  |  |  |  |  |  |  |  |  |  |  |  | 1 |
| Phaseshift Rapid In-vivo Measuring of human Skin (PRIMOS) ^16,183^ | | 2 | 1 |  |  |  |  |  |  |  | 2 |  |  |  |  |  |  |  |  |
| Surface profilometry ^9^ | | 1 | 1 |  |  |  |  |  |  |  | 1 |  |  |  |  |  |  |  |  |
| Combined devices | |  |  |  |  |  |  |  |  |  |  |  |  |  |  |  |  |  |  |
| DermaLab ^129,142^ | | 2 | 1 |  |  | 2 |  |  |  |  |  |  |  |  |  |  |  |  |  |
| DermaLab Combo ^77,194^ | | 2 | 2-5 |  |  | 2 |  | 2 |  | 2 |  | 1 |  |  |  |  | 1 |  |  |
| **Total** | | 148 | 1-5 | **49** | 2 | **28** | 3 | **27** | 10 | 17 | 9 | 5 | 1 | 5 | 4 | 6 | 1 | 2 | 2 |

1. Zhang CQ, Gogal C, Gaugler T, Blome-Eberwein S. A six-year experience of Laser Treatments for Burn Scars in a regional Burn Center-safety, efficacy and quality improvement. doi:10.1093/jbcr/iraa118/5873467

2. DCunha AR, Jehangir S, Rebekah G, Thomas RJ. Human Amniotic Membrane vs Collagen in the Treatment of Superficial Second-degree Burns in Children. *Wounds*. 2022;34(5):135-140. doi:10.25270/wnds/2022.135140

3. Fujiwara M, Suzuki T, Fukamizu H, Tokura Y. Successful treatment of postburn flexion contracture in fingers of early childhood with dynamic splint therapy after operation: long-term follow-up. *Eur J Plast Surg*. 2017;40(6):555-562. doi:10.1007/s00238-017-1288-9

4. Bailey JK, Burkes SA, Visscher MO, et al. Multimodal quantitative analysis of early pulsed-dye laser treatment of scars at a pediatric burn hospital. *Dermatologic Surgery*. 2012;38(9):1490-1496. doi:10.1111/j.1524-4725.2012.02451.x

5. Gurbuz K, Demir M, Das K. The Use of Dermal Substitute in Deep Burns of Functional/Mobile Anatomic Areas at Acute Phase After Early Excision and Subsequent Skin Autografting: Dermal Substitute Prevents Functional Limitations. doi:10.1093/jbcr/iraa047/5810790

6. Karlsson M, Steinvall I, Sjöberg F, Olofsson P, Elmasry M. Burn scar outcome at six and 12 months after injury in children with partial thickness scalds: Effects of dressing treatment. *Burns*. 2020;46(3):546-551. doi:10.1016/j.burns.2020.02.007

7. Cox C, Bettiol P, Le A, MacKay BJ, Griswold J, McKee D. CO 2 laser resurfacing for burn and traumatic scars of the hand and upper extremity . *Scars Burn Heal*. 2022;8:205951312110476. doi:10.1177/20595131211047694

8. Lau JCM, Li-Tsang CWP, Zheng YP. Application of tissue ultrasound palpation system (TUPS) in objective scar evaluation. *Burns*. 2005;31(4):445-452. doi:10.1016/j.burns.2004.07.016

9. Allison KP, Kiernan MN, Waters RA, Clement RM. Pulsed dye laser treatment of burn scars: Alleviation or irritation? *Burns*. 2003;29(3):207-213. doi:10.1016/S0305-4179(02)00280-2

10. Kubiak R, Lange B. Percutaneous collagen induction as an additive treatment for scar formation following thermal injuries: Preliminary experience in 47 children. *Burns*. 2017;43(5):1097-1102. doi:10.1016/j.burns.2017.02.006

11. Demircan M, Cicek T, Yetis MI. Preliminary results in single-step wound closure procedure of full-thickness facial burns in children by using the collagen-elastin matrix and review of pediatric facial burns. *Burns*. 2015;41(6):1268-1274. doi:10.1016/j.burns.2015.01.007

12. Lee SZ, Halim AS. Superior long term functional and scar outcome of Meek micrografting compared to conventional split thickness skin grafting in the management of burns. *Burns*. 2019;45(6):1386-1400. doi:10.1016/j.burns.2019.04.011

13. Kono T, Erçöçen AR, Nakazawa H, Nozaki M. Treatment of hypertrophic scars using a long-pulsed dye laser with cryogen-spray cooling. *Ann Plast Surg*. 2005;54(5):487-493. doi:10.1097/01.sap.0000155276.93061.93

14. Xiao Y, Sun Y, Zhu B, et al. Risk factors for hypertrophic burn scar pain, pruritus, and paresthesia development. *Wound Repair and Regeneration*. 2018;26(2):172-181. doi:10.1111/wrr.12637

15. Callcut RA, Schurr MJ, Sloan M, Faucher LD. Clinical experience with Alloderm: A one-staged composite dermal/epidermal replacement utilizing processed cadaver dermis and thin autografts. *Burns*. 2006;32(5):583-588. doi:10.1016/j.burns.2005.12.002

16. Poetschke J, Dornseifer U, Clementoni MT, et al. Ultrapulsed fractional ablative carbon dioxide laser treatment of hypertrophic burn scars: evaluation of an in-patient controlled, standardized treatment approach. *Lasers Med Sci*. 2017;32(5):1031-1040. doi:10.1007/s10103-017-2204-z

17. Elrod J, Schiestl C, Neuhaus D, Mohr C, Neuhaus K. Patient- And physician-reported outcome of combined fractional CO2and pulse dye laser treatment for hypertrophic scars in children. *Ann Plast Surg*. 2020;85(3):237-244. doi:10.1097/SAP.0000000000002377

18. Chinaroonchai K, Muangman P. *Case Series: New, Effective, Treatment Choice for Post-Burn Pruritus*. http://www.jmatonline.com

19. Ayaz M, Keshavarzi A, Bahadoran H, Arasteh P, Moslemi S. Comparison of the Results of Early Excision and Grafting between Children and Adults; A Prospective Comparative Study. *Bull Emerg Trauma*. 2017;5(3):179-183.

20. Boyce ST, Kagan RJ, Greenhalgh DG, et al. Cultured skin substitutes reduce requirements for harvesting of skin autograft for closure of excised, full-thickness burns. *J Trauma*. 2006;60(4):821-829. doi:https://doi.org/10.1097/01.ta.0000196802.91829.cc

21. Cattelaens J, Turco L, Berclaz LM, et al. The impact of a nanocellulose-based wound dressing in the management of thermal injuries in children: Results of a retrospective evaluation. *Life*. 2020;10(9):1-11. doi:10.3390/life10090212

22. Elrashid NAA, Sanad DA, Mahmoud NF, Hamada HA, Abdelmoety AM, Kenawy AM. *Effect of Orange Polarized Light on Post Burn Pediatric Scar: A Single Blind Randomized Clinical Trial*.

23. Elrod J, Moellmeier D, Schiestl C, Mohr C, Neuhaus K. Comparative analysis of functional and aesthetic outcomes of retroauricular full thickness versus plantar glabrous split thickness skin grafts in pediatric palmar hand burns. *Burns*. 2020;46(3):639-646. doi:10.1016/j.burns.2019.09.004

24. El-Zawahry BM, Sobhi RM, Bassiouny DA, Tabak SA. Ablative CO2 fractional resurfacing in treatment of thermal burn scars: an open-label controlled clinical and histopathological study. *J Cosmet Dermatol*. 2015;14(4):324-331. doi:https://doi.org/10.1111/jocd.12163

25. Gurbuz K, Demir M. Clinical Profile and Acute-Phase Management Modalities of Pediatric Hand Burn: A Retrospective Study. *EUROPEAN BURN JOURNAL*. 2022;3(1):34-42. doi:https://doi.org/10.3390/ebj3010005

26. Hamanová H, Broz L. Influence of inadequate prehospital and primary hospital treatment on the maturation of scars after thermal injuries. *Acta Chir Plast*. 2003;45(1):18-21.

27. Koller R, Kargül G, Giovanoli P, Meissl G, Frey M. Quantification of functional results after facial burns by the faciometer. *Burns*. 2000;26(8):716-723. doi:https://doi.org/10.1016/s0305-4179(00)00053-x

28. Karagoz H, Yuksel F, Ulkur E, Evinc R. Comparison of efficacy of silicone gel, silicone gel sheeting, and topical onion extract including heparin and allantoin for the treatment of postburn hypertrophic scars. *Burns*. 2009;35(8):1097-1103. doi:https://doi.org/10.1016/j.burns.2009.06.206

29. Kazemzadeh J, Yousefiazar A, Zahedi A. Amniotic membrane dressing versus nitrofurazone-impregnated dressing in the treatment of second-degree burn wounds: a randomized clinical trial. *Wounds*. 2021;34(1):11-16. doi:https://doi.org/10.25270/wnds/082421.04

30. Liang H, Hu Q, Cai J, et al. Functional and aesthetic outcomes of abdominal full-thickness skin grafts in paediatric postburn digital and palmar flexion contractures. *Int Wound J*. 2023;20(7):2718-2725. doi:https://doi.org/10.1111/iwj.14145

31. Matuszczak E, Weremijewicz A, Koper-Lenkiewicz OM, et al. Effects of combined Pulsed Dye Laser and Fractional CO2 Laser treatment of burn scars and correlation with plasma levels of collagen type I, MMP-2 and TIMP-1. *Burns*. 2021;47(6):1342-1351. doi:10.1016/j.burns.2020.12.011

32. Pham TD, Karlsson M, Andersson CM, Mirdell R, Sjoberg F. Automated VSS-based Burn Scar Assessment using Combined Texture and Color Features of Digital Images in Error-Correcting Output Coding. *Sci Rep*. 2017;7(1). doi:10.1038/s41598-017-16914-0

33. Nikkonen MM, Pitkanen JM, Al-Qattan MM. Problems associated with the use of silicone gel sheeting for hypertrophic scars in the hot climate of Saudi Arabia. *Burns*. 2001;27(5):498-501. doi:https://doi.org/10.1016/s0305-4179(01)00004-3

34. Sheridan K, Choucair R, Donelan M, Lydon M, Petras L, Tompkins R. Acellular allodermis in burn surgery: 1-year results of a pilot trial. *Journal of Burn Care and Rehabilitation*. 1998;19(6):528-530. doi:https://doi.org/10.1097/00004630-199811000-00012

35. Sheridan RL, MacMillan K, Donelan M, et al. Tunable dye laser neovessel ablation as an adjunct to the management of hypertrophic scarring in burned children: pilot trial to establish safety. *J Burn Care Rehabil*. 1997;18(4):317-320. doi:https://doi.org/10.1097/00004630-199707000-00007

36. Sheridan RL, Lydon MM, Petras LM, et al. Laser ablation of burns: initial clinical trial. *Surgery*. 1999;125(1):92-95. doi:https://doi.org/10.1016/s0039-6060(99)70293-x

37. Šín P, Brychta P. Cutometrical measurement confirms the efficacy of the composite skin grafting using allogeneic acellular dermis in burns. *Acta Chir Plast*. 2006;48(2):59-64. https://www.embase.com/search/results?subaction=viewrecord&id=L44355521&from=export

38. Wala SJ, Patterson K, Scoville S, et al. A single institution case series of ReCell(®) use in treating pediatric burns. *Int J Burns Trauma*. 2023;13(2):78-88.

39. Vloemans A, Soesman AM, Kreis RW, Middelkoop E. A newly developed hydrofibre dressing, in the treatment of partial-thickness burns. *BURNS*. 2001;27(2):167-173. doi:https://doi.org/10.1016/s0305-4179(00)00080-2

40. Waymack P, Duff RG, Sabolinski M. The effect of a tissue engineered bilayered living skin analog, over meshed split-thickness autografts on the healing of excised burn wounds. The Apligraf Burn Study Group. *Burns*. 2000;26(7):609-619. doi:https://doi.org/10.1016/s0305-4179(00)00017-6

41. Weshahy RH, Aly DG, Shalaby S, Mohammed FN, Sayed KS. Clinical and Histological Assessment of Combined Fractional CO2 Laser and Growth Factors Versus Fractional CO2 Laser Alone in the Treatment of Facial Mature Burn Scars: A Pilot Split-Face Study. *Lasers Surg Med*. 2020;52(10):952-958. doi:https://doi.org/10.1002/lsm.23252

42. Wood AJ, Clugston SC, Rawlins JM, Rea S, Edgar DW, Wood FM. Burn patients, parents and doctors; are we in agreement? *Burns*. 2012;38(4):487-492. doi:https://doi.org/10.1016/j.burns.2012.01.004

43. Wyrzykowski D, Chrzanowska B, Czauderna P. Ten years later-scalp still a primary donor site in children. *Burns*. 2015;41(2):359-363. doi:https://doi.org/10.1016/j.burns.2014.07.009

44. Yagmur Ç, Evin N, Engin MS, Simsek T, Küçüker I, Demir A. Post-Burn Skin Deformities of the Face and Neck Region in Pediatric Patients: Single-Stage Treatment Using Collagen Elastin Matrix. *TURKISH JOURNAL OF PLASTIC SURGERY*. 2017;25(3):126-131. doi:https://doi.org/10.5152/turkjplastsurg.2017.2161

45. Zayed AA, Mashaly HM, Abdel Raheem HM, et al. Microneedling versus fractional CO2 laser in the treatment of atrophic postburn scars. *Journal of the Egyptian Women’s Dermatologic Society*. 2019;16(1):37-42. doi:https://doi.org/10.4103/jewd.jewd_1_19

46. Thomas R, Wicks S, Dale M, Pacey V. Outcomes of Early and Intensive Use of a Palm and Digit Extension Orthosis in Young Children After Burn Injury. *Journal of Burn Care and Research*. 2021;42(2):245-257. doi:10.1093/jbcr/iraa137

47. Fraulin FO, Illmayer SJ, Tredget EE. Assessment of cosmetic and functional results of conservative versus surgical management of facial burns. *J Burn Care Rehabil*. 1996;17(1):19-29. doi:https://doi.org/10.1097/00004630-199601000-00007

48. Argirova M, Hadjiski O, Victorova A. Non-operative treatment of hypertrophic scars and keloids after burns in children. *Ann Burns Fire Disasters*. 2006;19(2):80-87.

49. Sullivan T, Smith J, Kermode J, McIver E, Courtemanche DJ. Rating the burn scar. *J Burn Care Rehabil*. 1990;11(3):256-260. doi:https://doi.org/10.1097/00004630-199005000-00014

50. Chou TD, Chen SL, Lee TW, et al. Reconstruction of burn scar of the upper extremities with artificial skin. *Plast Reconstr Surg*. 2001;108(2):378-384. doi:https://doi.org/10.1097/00006534-200108000-00015

51. Majid I, Imran S. Fractional Carbon Dioxide Laser Resurfacing in Combination With Potent Topical Corticosteroids for Hypertrophic Burn Scars in the Pediatric Age Group: An Open Label Study. *Dermatol Surg*. 2018;44(8):1102-1108. doi:https://doi.org/10.1097/dss.0000000000001413

52. de Oliveira G V, Sanford AP, Murphy KD, et al. Growth hormone effects on hypertrophic scar formation: a randomized controlled trial of 62 burned children. *Wound Repair Regen*. 2004;12(4):404-411. doi:https://doi.org/10.1111/j.1067-1927.2004.012407.x

53. Li X, Meng X, Wang X, et al. Human acellular dermal matrix allograft: A randomized, controlled human trial for the long-term evaluation of patients with extensive burns. *Burns*. 2015;41(4):689-699. doi:10.1016/j.burns.2014.12.007

54. El-Hoshy K, Abdel-Halim MRE, Dorgham D, El-Din Sayed SS, El-Kalioby M. Efficacy of fractional carbon dioxide laser in the treatment of mature burn scars: A clinical, histopathological, and histochemical study. *Journal of Clinical and Aesthetic Dermatology*. 2017;10(12):36-43. https://www.embase.com/search/results?subaction=viewrecord&id=L620029193&from=export

55. Nieuwendijk SMP, de Korte IJ, Pursad MM, van Dijk M, Rode H. Post burn pruritus in pediatric burn patients. *BURNS*. 2018;44(5):1151-1158. doi:https://doi.org/10.1016/j.burns.2018.02.022

56. Wei Y, Li-Tsang CWP, Luk DCK, Tan T, Zhang W, Chiu TW. A validation study of scar vascularity and pigmentation assessment using dermoscopy. *Burns*. 2015;41(8):1717-1723. doi:10.1016/j.burns.2015.05.013

57. Valladares-Poveda S, Avendaño-Leal O, Castillo-Hidalgo H, Murillo E, Palma C, Parry I. A comparison of two scar massage protocols in pediatric burn survivors. *Burns*. 2020;46(8):1867-1874. doi:10.1016/j.burns.2020.05.013

58. Sarkar A, Dewangan YK, Bain J, et al. Effect of intense pulsed light on immature burn scars: A clinical study. *Indian Journal of Plastic Surgery*. 2014;47(3):381-385. doi:10.4103/0970-0358.146596

59. Bowes LE, Nouri K, Berman B, et al. Treatment of pigmented hypertrophic scars with the 585 nm pulsed dye laser and the 532 nm frequency-doubled Nd:YAG laser in the Q-switched and variable pulse modes: a comparative study. *Dermatol Surg*. 2002;28(8):714-719. doi:https://doi.org/10.1046/j.1524-4725.2002.01058.x

60. Schwanholt CA, Ridgway CL, Greenhalgh DG, et al. A prospective study of burn scar maturation in pediatrics: does age matter? *J Burn Care Rehabil*. 1994;15(5):416-420. doi:https://doi.org/10.1097/00004630-199409000-00007

61. Gibbons M, Zuker R, Brown M, Candlish S, Snider L, Zimmer P. Experience with silastic gel sheeting in pediatric scarring. *J Burn Care Rehabil*. 1994;15(1):69-73. doi:https://doi.org/10.1097/00004630-199401000-00013

62. Armendariz-Borunda J, Lyra-Gonzalez I, Medina-Preciado D, et al. A controlled clinical trial with pirfenidone in the treatment of pathological skin scarring caused by burns in pediatric patients. *Ann Plast Surg*. 2012;68(1):22-28. doi:10.1097/SAP.0b013e31821b6d08

63. Vloemans AFPM, Soesman AM, Suijker M, Kreis RW, Middelkoop E. A randomised clinical trial comparing a hydrocolloid-derived dressing and glycerol preserved allograft skin in the management of partial thickness burns. *Burns*. 2003;29(7):702-710. doi:10.1016/S0305-4179(03)00161-X

64. Akita S, Akino K, Imaizumi T, et al. The quality of pediatric burn scars is improved by early administration of basic fibroblast growth factor. *Journal of Burn Care and Research*. 2006;27(3):333-338. doi:10.1097/01.BCR.0000216742.23127.7A

65. Harte D, Gordon J, Shaw M, Stinson M, Porter-Armstrong A. The use of pressure and silicone in hypertrophic scar management in burns patients: A pilot randomized controlled trial. *Journal of Burn Care and Research*. 2009;30(4):632-642. doi:10.1097/BCR.0b013e3181ac01a3

66. Parry I, Sen S, Palmieri T, Greenhalgh D. Nonsurgical scar management of the face: Does early versus late intervention affect outcome? *Journal of Burn Care and Research*. 2013;34(5):569-575. doi:10.1097/BCR.0b013e318278906d

67. Hayashida K, Fujioka M, Morooka S, Saijo H, Akita S. Effectiveness of basic fibroblast growth factor for pediatric hand burns. *J Tissue Viability*. 2016;25(4):220-224. doi:10.1016/j.jtv.2016.06.007

68. Draaijers LJ, Tempelman FRH, Botman YAM, et al. The Patient and Observer Scar Assessment Scale: A reliable and feasible tool for scar evaluation. *Plast Reconstr Surg*. 2004;113(7):1960-1965. doi:10.1097/01.PRS.0000122207.28773.56

69. Ma Z, Mo R, Chen C, Meng X, Tan Q. Surgical treatment of joint burn scar contracture: a 10-year single-center experience with long-term outcome evaluation. *Ann Transl Med*. 2021;9(4):303-303. doi:10.21037/atm-20-4947

70. Moiemen N, Mathers J, Jones L, et al. Pressure garment to prevent abnormal scarring after burn injury in adults and children: The PEGASUS feasibility RCT and mixed-methods study. *Health Technol Assess (Rockv)*. 2018;22(36):1-162. doi:10.3310/hta22360

71. Palao R, Gómez PA, Huguet P. Burned breast reconstructive surgery with integra dermal regeneration template. *Br J Plast Surg*. 2003;56(3):252-259. doi:10.1016/S0007-1226(03)00101-2

72. Yu P xi, Diao W qi, Qi Z liang, Cai J long. Effect of Dermabrasion and ReCell® on Large Superficial Facial Scars Caused by Burn, Trauma and Acnes. *Chinese Medical Sciences Journal*. 2016;31(3):173-179. doi:10.1016/S1001-9294(16)30047-5

73. Oh SJ, Kim Y. Combined AlloDerm® and thin skin grafting for the treatment of postburn dyspigmented scar contracture of the upper extremity. *Journal of Plastic, Reconstructive and Aesthetic Surgery*. 2011;64(2):229-233. doi:10.1016/j.bjps.2010.04.017

74. Finlay V, Burrows S, Kendell R, et al. Modified Vancouver Scar Scale score is linked with quality of life after burn. *Burns*. 2017;43(4):741-746. doi:10.1016/j.burns.2016.11.007

75. Válik A, Harangozó K, Garami A, Juhász Z, Józsa G, Lőrincz A. Mid-Term Follow-Up Study of Children Undergoing Autologous Skin Transplantation for Burns. *Life*. 2023;13(3). doi:10.3390/life13030762

76. Zadkowski T, Nachulewicz P, Mazgaj M, et al. A new CO2 laser technique for the treatment of pediatric hypertrophic burn scars: An observational study. *Medicine (United States)*. 2016;95(42). doi:10.1097/MD.0000000000005168

77. Zuccaro J, Kelly C, Perez M, Doria A, Fish JS. The effectiveness of laser therapy for hypertrophic burn scars in pediatric patients: A prospective investigation. In: *Journal of Burn Care and Research*. Vol 42. Oxford University Press; 2021:847-856. doi:10.1093/jbcr/irab090

78. Zuccaro J, Muser I, Singh M, Yu J, Kelly C, Fish J. Laser Therapy for Pediatric Burn Scars: Focusing on a Combined Treatment Approach. *Journal of Burn Care and Research*. 2018;39(3):457-462. doi:10.1093/jbcr/irx008

79. Fraccalvieri M, Sarno A, Gasperini S, et al. *Can Single Use Negative Pressure Wound Therapy Be an Alternative Method to Manage Keloid Scarring? A Preliminary Report of a Clinical and Ultrasound/Colour-Power-Doppler Study*.; 2012.

80. Hayashida K, Akita S. *Quality of Pediatric Second-Degree Burn Wound Scars Following the Application of Basic Fibroblast Growth Factor: Results of a Randomized’ Controlled Pilot Study*.; 2014. https://www.researchgate.net/publication/230643432

81. Khatery BHM, Hussein HA, Abd-el-Raheem TA, El Hanbuli HM, Yassen NN. Assessment of intralesional injection of botulinum toxin type A in hypertrophic scars and keloids: Clinical and pathological study. *Dermatol Ther*. 2022;35(10). doi:10.1111/dth.15748

82. Hamanová H, Broz L. Topigel in the treatment of hypertrophic scars after burn injuries. *Acta Chir Plast*. 2002;44(1):18-22.

83. Hartmann B, Ekkernkamp A, Johnen C, Gerlach JC, Belfekroun C, Küntscher M V. Sprayed cultured epithelial autografts for deep dermal burns of the face and neck. *Ann Plast Surg*. 2007;58(1):70-73. doi:https://doi.org/10.1097/01.sap.0000250647.39784.bb

84. Gravante G, Sorge R, Merone A, et al. Hyalomatrix PA in burn care practice: results from a national retrospective survey, 2005 to 2006. *Ann Plast Surg*. 2010;64(1):69-79. doi:https://doi.org/10.1097/sap.0b013e31819b3d59

85. Jiang Y, Guo R, Zhou S, Wang B, Sun W. Functional and aesthetic reconstruction of digital flexion contractures with full-thickness plantar skin grafts in children. *Dermatol Ther*. 2020;33(6):e14466. doi:https://doi.org/10.1111/dth.14466

86. Lang EM, Eiberg CA, Brandis M, Stark GB. Biobrane in the treatment of burn and scald injuries in children. *Ann Plast Surg*. 2005;55(5):485-489. doi:https://doi.org/10.1097/01.sap.0000182652.88669.a6

87. Luo B, Sun HT, Wang YT, et al. Clinical efficacy of rhGM-CSF gel and medical collagen sponge on deep second-degree burns of infants: A randomized clinical trial. *Medicine (United States)*. 2024;103(1):E36304. doi:10.1097/MD.0000000000036304

88. Rab M, Koller R, Ruzicka M, et al. Should dermal scald burns in children be covered with autologous skin grafts or with allogeneic cultivated keratinocytes?–"The Viennese concept". *Burns*. 2005;31(5):578-586. doi:https://doi.org/10.1016/j.burns.2005.01.001

89. Ueda M. Sprayed cultured mucosal epithelial cell for deep dermal burns. *J Craniofac Surg*. 2010;21(6):1729-1732. doi:https://doi.org/10.1097/scs.0b013e3181f3c78b

90. Yao Z, Peng M, Liao J, et al. Treatment of upper eyelid third-degree burns by dispersed implantation of very small autologous columnar skin grafts: A pilot study of a new method. *Burns*. 2022;48(7):1671-1679. doi:https://doi.org/10.1016/j.burns.2022.01.011

91. Zuccaro J, Perez MM, Mohanta A, Fish JS, Doria AS. Quantification of Pediatric Burn Scar Stiffness Using Acoustic Radiation Force Impulse Ultrasound Elastography. *Ultrasound Med Biol*. 2019;45(8):1918-1923. doi:10.1016/j.ultrasmedbio.2019.03.013

92. Brown CA. The use of silicon gel for treating children’s burn scars in Saudi Arabia: a case study. *Occup Ther Int*. 2002;9(2):121-130. doi:https://doi.org/10.1002/oti.160

93. Elmelegy NG, Hegazy AM, Sadaka MS, Abdeldaim DE. Electrophotobiomodulation in the treatment of facial post-burn hypertrophic scars in pediatric patients. *Ann Burns Fire Disasters*. 2018;31(2):127-132.

94. Oliveira G V, Chinkes D, Mitchell C, Oliveras G, Hawkins HK, Herndon DN. Objective assessment of burn scar vascularity, erythema, pliability, thickness, and planimetry. *Dermatol Surg*. 2005;31(1):48-58. doi:https://doi.org/10.1097/00042728-200501000-00010    https://doi.org/10.1111/j.1524-4725.2005.31004

95. Juhasz I, Kiss B, Lukacs L, Erdei I, Peter Z, Remenyik E. Long-term followup of dermal substitution with acellular dermal implant in burns and postburn scar corrections. *Dermatol Res Pract*. 2010;2010(1). doi:10.1155/2010/210150

96. Draaijers LJ, Tempelman FRH, Botman YAM, Kreis RW, Middelkoop E, Van Zuijlen PPM. Colour evaluation in scars: Tristimulus colorimeter, narrow-band simple reflectance meter or subjective evaluation? *Burns*. 2004;30(2):103-107. doi:10.1016/j.burns.2003.09.029

97. Thomas R, Stephanie Wicks M, Sci Claire Toose Ba, Verity Pacey Ba, Cert Sports Phty G. Title: Outcomes of early use of an end of range axilla orthotic in children following burn injury. Published online 2019. doi:10.1093/jbcr/irz058/5428608

98. Wallace HJ, Fear MW, Crowe MM, Martin LJ, Wood FM. Identification of factors predicting scar outcome after burn injury in children: A prospective case-control study. *Burns Trauma*. 2017;5(1). doi:10.1186/s41038-017-0084-x

99. Wallace HJ, Cadby G, Melton PE, et al. Genetic influence on scar height and pliability after burn injury in individuals of European ancestry: A prospective cohort study. *Burns*. 2019;45(3):567-578. doi:10.1016/j.burns.2018.10.027

100. Vehmeyer-Heeman M, Tondu T, den Kerckhove E, Boeckx W. Application of cerium nitrate-silver sulphadiazine allows for postponement of excision and grafting. *Burns*. 2006;32(1):60-63. doi:https://doi.org/10.1016/j.burns.2005.06.022

101. Hyland EJ, D’Cruz R, Menon S, et al. Prospective, randomised controlled trial comparing Versajet^TM^ hydrosurgery and conventional debridement of partial thickness paediatric burns. *Burns*. 2015;41(4):700-707. doi:10.1016/j.burns.2015.02.001

102. Choi YH, Kim KM, Kim HO, Jang YC, Kwak IS. Clinical and histological correlation in post-Burn hypertrophic scar for pain and itching sensation. *Ann Dermatol*. 2013;25(4):428-433. doi:10.5021/ad.2013.25.4.428

103. Muangman P, Aramwit P, Palapinyo S, Opasanon S, Chuangsuwanich A. Efficacy of the combination of herbal extracts and a silicone derivative in the treatment of hypertrophic scar formation after burn injury. *Afr J Pharm Pharmacol*. 2011;5(3):442-446. doi:10.5897/AJPP10.282

104. Kawecki M, Bernad-Wiśniewska T, Sakiel S, Nowak M, Andriessen A. Laser in the treatment of hypertrophic burn scars. *Int Wound J*. 2008;5(1):87-97. doi:https://doi.org/10.1111/j.1742-481x.2007.00309.x

105. Herndon D, Capek KD, Ross E, et al. Reduced postburn hypertrophic scarring and improved physical recovery with yearlong administration of oxandrolone and propranolol. *Ann Surg*. 2018;268(3):431-441. doi:10.1097/SLA.0000000000002926

106. Nedelec B, Shankowsky H, Tredget E. Rating the resolving hypertrophic scar: Comparison of the Vancouver Scar Scale and scar volume. *Journal of Burn Care and Rehabilitation*. 2000;21(3):205-212.

107. Rosenberg L, Krieger Y, Bogdanov-Berezovski A, Silberstein E, Shoham Y, Singer AJ. A novel rapid and selective enzymatic debridement agent for burn wound management: A multi-center RCT. *Burns*. 2014;40(3):466-474. doi:10.1016/j.burns.2013.08.013

108. Chipp E, Charles L, Thomas C, Whiting K, Moiemen N, Wilson Y. A prospective study of time to healing and hypertrophic scarring in paediatric burns: Every day counts. *Burns Trauma*. 2017;5(1). doi:10.1186/s41038-016-0068-2

109. Momeni M, Hafezi F, Rahbar H, Karimi H. Effects of silicone gel on burn scars. *Burns*. 2009;35(1):70-74. doi:10.1016/j.burns.2008.04.011

110. Scott Hultman C, Friedstat JS, Edkins RE, Cairns BA, Meyer AA. Laser resurfacing and remodeling of hypertrophic burn scars: The results of a large, prospective, before-After cohort study, with long-term follow-up. In: *Annals of Surgery*. Vol 260. Lippincott Williams and Wilkins; 2014:519-532. doi:10.1097/SLA.0000000000000893

111. Lee JH, Seo CE, Song WJ, et al. Combination treatment utilizing fractional ablative and continuous wave CO2 lasers for hypertrophic burn scars. *Burns*. 2021;47(5):1084-1093. doi:10.1016/j.burns.2020.10.015

112. Lei Y, Li SF, Yu YL, Tan J, Gold MH. Clinical efficacy of utilizing Ultrapulse CO2 combined with fractional CO2 laser for the treatment of hypertrophic scars in Asians—A prospective clinical evaluation. *J Cosmet Dermatol*. 2017;16(2):210-216. doi:10.1111/jocd.12334

113. Ouyang HW, Li GF, Lei Y, Gold MH, Tan J. Comparison of the effectiveness of pulsed dye laser vs pulsed dye laser combined with ultrapulse fractional CO2 laser in the treatment of immature red hypertrophic scars. *J Cosmet Dermatol*. 2018;17(1):54-60. doi:10.1111/jocd.12487

114. Yelvington M, Scoggins M, White L. A child with a burn-related foot and ankle contracture treated with multiple modalities. *Pediatric Physical Therapy*. 2017;29(1):90-94. doi:10.1097/PEP.0000000000000346

115. Li N, Yang L, Cheng J, et al. A retrospective study to identify the optimal parameters for pulsed dye laser in the treatment of hypertrophic burn scars in Chinese children with Fitzpatrick skin types III and IV. *Lasers Med Sci*. 2021;36(8):1671-1679. doi:10.1007/s10103-021-03252-x

116. Li N, Yang L, Cheng J, Han J, Hu D. Early intervention by Z-plasty combined with fractional CO(2) laser therapy as a potential treatment for hypertrophic burn scars. *J Plast Reconstr Aesthet Surg*. 2021;74(11):3087-3093. doi:https://doi.org/10.1016/j.bjps.2021.03.079

117. Yelvington M, Brown S, Castro MM, Nick TG. The use of neoprene as a scar management modality. *Burns*. 2013;39(5):866-875. doi:https://doi.org/10.1016/j.burns.2012.11.002

118. Liu H, Shu F, Ji C, et al. Clarifying sleep characteristics and analyzing risk factors of sleep disorders to promote a predictive, preventive, and personalized medicine in patients with burn scars. *EPMA Journal*. 2023;14(1):131-142. doi:10.1007/s13167-022-00309-x

119. Moiemen NS, Vlachou E, Staiano JJ, Thawy Y, Frame JD. Reconstructive surgery with integra dermal regeneration template: Histologic study, clinical evaluation, and current practice. *Plast Reconstr Surg*. 2006;117(7 SUPPL.). doi:10.1097/01.prs.0000222609.40461.68

120. Kawecki M, Bernad-Wis T, Sakiel S, Nowak M, Andriessen Kawecki AM, Laser AA. *Between 21*.; 2000.

121. Alsharnoubi J, Shoukry KES, Fawzy MW, Mohamed O. Evaluation of scars in children after treatment with low-level laser. *Lasers Med Sci*. 2018;33(9):1991-1995. doi:10.1007/s10103-018-2572-z

122. Simons M, Ziviani J, Thorley M, McNee J, Tyack Z. Exploring reliability of scar rating scales using photographs of burns from children aged up to 15 years. *J Burn Care Res*. 2013;34(4):427-438. doi:https://doi.org/10.1097/bcr.0b013e3182700054

123. Forbes-Duchart L, Marshall S, Strock A, Cooper JE. Determination of inter-rater reliability in pediatric burn scar assessment using a modified version of the vancouver scar scale. *Journal of Burn Care and Research*. 2007;28(3):460-467. doi:10.1097/BCR.0b013E318053D3BB

124. Jaworski W, Jaworski A, Kufel J, Grabowski A, Korlacki W. Deep and extensive pediatric burns: retrospective evaluation of scheme of patients at the Department of Children’s Developmental Defects Surgery and Traumatology SUM. *Polish Journal of Surgery*. 2022;94(5):31-39. doi:10.5604/01.3001.0015.7679

125. Vana LPM, Battlehner CN, Ferreira MA, Caldini EG, Gemperli R, Alonso N. Comparative long-term study between two dermal regeneration templates for the reconstruction of burn scar contractures in humans: Clinical and histological results. *Burns*. 2020;46(3):596-608. doi:10.1016/j.burns.2019.09.005

126. Gal S, Ramirez JI, Maguina P. Autologous fat grafting does not improve burn scar appearance: A prospective, randomized, double-blinded, placebo-controlled, pilot study. *Burns*. 2017;43(3):486-489. doi:10.1016/j.burns.2016.09.019

127. Gnipov PA, Baindurashvili AG, Brazol MA, Mitrofanova E V., Melnikov MR, Mashevskiy GA. The benefits of early surgical treatment of deep cervical burns in children. *Pediatric Traumatology, Orthopaedics and Reconstructive Surgery*. 2020;8(1):25-34. doi:10.17816/PTORS16298

128. Al-Mohamady AESAEH, Ibrahim SMA, Muhammad MM. Pulsed dye laser versus long-pulsed Nd:YAG laser in the treatment of hypertrophic scars and keloid: A comparative randomized split-scar trial. *Journal of Cosmetic and Laser Therapy*. 2016;18(4):208-212. doi:10.3109/14764172.2015.1114648

129. Staubach R, Glosse H, Fennell S, Loff S. A Single-Institution Experience about 10 Years with Children Undergoing Fractional Ablative Carbon Dioxide Laser Treatment after Burns: Measurement of Air Pressure-Induced Skin Elevation and Retraction Time (Dermalab) Including Standardized Subjective and Objective Scar Evaluation. *Journal of Burn Care and Research*. 2023;44(3):655-669. doi:10.1093/jbcr/irac125

130. Wang J, Wu J, Xu M, et al. A comprehensive reconstruction strategy for moderate to severe faciocervical scar contractures. *Lasers Med Sci*. 2021;36(6):1275-1282. doi:10.1007/s10103-020-03178-w

131. Seo DK, Kym D, Hur J. Management of neck contractures by single-stage dermal substitutes and skin grafting in extensive burn patients. *Ann Surg Treat Res*. 2014;87(5):253-259. doi:10.4174/astr.2014.87.5.253

132. Patiño O, Novick C, Merlo A, Benaim F. Massage in hypertrophic scars. *J Burn Care Rehabil* . 1999;20(3):268-271+267.

133. Chan QE, Barzi F, Harvey JG, Holland AJA. Functional and cosmetic outcome of full- versus split-thickness skin grafts in pediatric palmar surface burns: A prospective, independent evaluation. *Journal of Burn Care and Research*. 2013;34(2):232-236. doi:10.1097/BCR.0b013e31826fc53d

134. Li-Tsang CWP, Lau JCM, Liu SKY. Validation of an objective scar pigmentation measurement by using a spectrocolorimeter. *Burns*. 2003;29(8):779-784. doi:10.1016/S0305-4179(03)00165-7

135. Ahuja N, Jin R, Powers C, Billi A, Bass K. Dehydrated Human Amnion Chorion Membrane as Treatment for Pediatric Burns. *Adv Wound Care (New Rochelle)*. 2020;9(11):602-611. doi:https://doi.org/10.1089/wound.2019.0983

136. Chen B, Song H. Retrospective study of the application of acellular dermis in reconstructing full-thickness skin defects. *Int Wound J*. 2017;14(1):158-164. doi:https://doi.org/10.1111/iwj.12576

137. Kivi MK, Jafarzadeh A, Hosseini-Baharanchi FS, Salehi S, Goodarzi A. The efficacy, satisfaction, and safety of carbon dioxide (CO2) fractional laser in combination with pulsed dye laser (PDL) versus each one alone in the treatment of hypertrophic burn scars: a single-blinded randomized controlled trial. *Lasers Med Sci*. 2024;39(1). doi:https://doi.org/10.1007/s10103-024-03976-6

138. Tawfik AA, Ali RA. Intense pulsed light in the management of post-burn keloid and hypertrophic scar: a comparative study. *Eur J Plast Surg*. 2023;46(6):1323-1329. doi:https://doi.org/10.1007/s00238-023-02103-y

139. Tawfik AA, Ali RA. Evaluation of botulinum toxin type A for treating post burn hypertrophic scars and keloid in children: An intra-patient randomized controlled study. *J Cosmet Dermatol*. 2023;22(4):1256-1260. doi:https://doi.org/10.1111/jocd.15634

140. Noordenbos J, Doré C, Hansbrough JF. Safety and efficacy of TransCyte for the treatment of partial-thickness burns. *J Burn Care Rehabil*. 1999;20(4):275-281. doi:https://doi.org/10.1097/00004630-199907000-00001    https://doi.org/10.1097/00004630-199907000-00002

141. Feng X, Tan J, Pan Y, et al. Control of hypertrophic scar from inception by using xenogenic (porcine) acellular dermal matrix (ADM) to cover deep second degree burn. *Burns*. 2006;32(3):293-298. doi:10.1016/j.burns.2005.10.006

142. Pirayesh A, Hoeksema H, Richters C, Verbelen J, Monstrey S. Glyaderm® dermal substitute: Clinical application and long-term results in 55 patients. *Burns*. 2015;41(1):132-144. doi:10.1016/j.burns.2014.05.013

143. Zhou Z, Sun X, Yin Z, et al. Comparative effectiveness analysis of deep low heat burns in the shin surgical approaches: Outcomes and cost for wound rehabilitation. *Int Wound J*. 2023;20(6):1832-1838. doi:https://doi.org/10.1111/iwj.14033

144. Delgado-Miguel C, Miguel-Ferrero M. Preliminary results of the use of carboxytherapy in the treatment of pathologic scars: A minimally invasive alternative. *J Pediatr Surg*. 2023;58(4):679-683. doi:10.1016/j.jpedsurg.2022.12.008

145. Cubison TCS, Pape SA, Parkhouse N. Evidence for the link between healing time and the development of hypertrophic scars (HTS) in paediatric burns due to scald injury. *Burns*. 2006;32(8):992-999. doi:10.1016/j.burns.2006.02.007

146. Kaźmierski M, Mańkowski P, Jankowski A, Harasymczuk J. Comparison of the results of operative and conservative treatment of deep dermal partial-thickness scalds in children. *European Journal of Pediatric Surgery*. 2007;17(5):354-361. doi:10.1055/s-2006-924646

147. Karimi H, Mobayen M, Alijanpour A. *STUDENT CORNER Management of Hypertrophic Burn Scar: A Comparison between the Efficacy of Exercise-Physiotherapy and Pressure Garment-Silicone on Hypertrophic Scar*. Vol 4.; 2013. http://asjsm.tums.ac.ir

148. Khandelwal A, Yelvington M, Tang X, Brown S. Ablative fractional photothermolysis for the treatment of hypertrophic burn scars in adult and pediatric patients: A single surgeon’s experience. *Journal of Burn Care and Research*. 2014;35(5):455-463. doi:10.1097/BCR.0000000000000028

149. Zhou R, Qiu L, Xiao J, Mao X, Yuan X. Early wound repair versus later scar repair in children with treadmill hand friction burns. Published online 2015. doi:10.1093/jbcr/irab083/6279077

150. Song Y, Li Y, Xia C, et al. Use of Tissue Expansion and Serial Z-plasty for Release of Neck Post-Burn Scar Contracture. Published online 2019. doi:10.1093/jbcr/irz134/5532827

151. Artzi O, Koren A, Niv R, Mehrabi JN, Mashiah J, Friedman O. A new approach in the treatment of pediatric hypertrophic burn scars: Tixel-associated topical triamcinolone acetonide and 5-fluorouracil delivery. *J Cosmet Dermatol*. 2020;19(1):131-134. doi:10.1111/jocd.13192

152. Chen L, Yang J, Wang D yun, et al. Multicenter effect analysis of one-step acellular dermis combined with autologous ultra-thin split thickness skin composite transplantation in treating burn and traumatic wounds. *Int Wound J*. 2024;21(1). doi:10.1111/iwj.14341

153. Danin A, Georgesco G, Le Touze A, Penaud A, Quignon R, Zakine G. Assessment of burned hands reconstructed with Integra® by ultrasonography and elastometry. *Burns*. 2012;38(7):998-1004. doi:https://doi.org/10.1016/j.burns.2012.02.017

154. Zhang ZB, Zhou ZL, Xing FX, et al. Analysis of Energy and Density in Treating Hypertrophic Scar After Burn in Children with CO(2) Dot Matrix Laser. *Int J Low Extrem Wounds*. Published online July 2022:15347346221144152. doi:https://doi.org/10.1177/15347346221144152

155. Zajíček R, Grossová I, Šuca H, Kubok R, Pafčuga I. EXPERIENCE WITH INTEGRA® AT THE PRAGUE BURNS CENTRE 2002-2016. *Acta Chir Plast*. 2017;59(1):18-26.

156. Wood F, Martin L, Lewis D, et al. A prospective randomised clinical pilot study to compare the effectiveness of Biobrane® synthetic wound dressing, with or without autologous cell suspension, to the local standard treatment regimen in paediatric scald injuries. *Burns*. 2012;38(6):830-839. doi:https://doi.org/10.1016/j.burns.2011.12.020

157. van Oudtshoorn S, Wood F, McWilliams T. Neonatal burns: a 10-year review of community- and hospital-acquired neonatal burns in Western Australia. *ANZ J Surg*. 2021;91(11):2503-2506. doi:https://doi.org/10.1111/ans.17198

158. Tushar M, Sandhyarani P, Jaganath SS, et al. Study of Clinical Response between Treatment of Keloids with Intralesional Injection of Bleomycin plus Triamcinolone Acetonide versus Intralesional Cryotherapy plus Triamcinolone Acetonide. *International Journal of Toxicological and Pharmacological Research*. 2024;14(1):119-125. https://www.embase.com/search/results?subaction=viewrecord&id=L2027873524&from=export

159. Tawfic S, Sayed S, Nada A, Manaa D, Shalaby S. High- Versus Low-Density Fractional Laser in the Treatment of Hypertrophic Postburn Scars: A Randomized Clinical Trial. *Dermatol Surg*. 2020;46(9):e38-e44. doi:https://doi.org/10.1097/dss.0000000000002293

160. Slocombe PD, Simons MA, Kimble RM. A modification of the Hynes procedure–a surgical innovation in the treatment of mature hypertrophic scars in children. *Burns*. 2011;37(7):1265-1267. doi:https://doi.org/10.1016/j.burns.2011.04.012

161. Ayaz M, Najafi A, Karami MY. Thin Split Thickness Skin Grafting On Human Acellular Dermal Matrix Scaffold For The Treatment Of Deep Burn Wounds. *Int J Organ Transplant Med*. 2021;12(1):44-51. https://www.embase.com/search/results?subaction=viewrecord&id=L2011153638&from=export

162. Joo HS, Lee SJ, Sung KY. Early Debridement and Cultured Allogenic Keratinocyte Dressing Prevent Hypertrophic Scarring in Infants with Deep Dermal Burns. *Archives of Aesthetic Plastic Surgery*. 2018;24(3):111-115. doi:10.14730/aaps.2018.24.3.111

163. Alsharnoubi J, Mohamed O. Photobiomodulation effect on children’s scars. *Lasers Med Sci*. 2018;33(3):497-501. doi:https://doi.org/10.1007/s10103-017-2387-3

164. Wood FM, Currie K, Backman B, Cena B. Current difficulties and the possible future directions in scar assessment. *Burns*. 1996;22(6):455-458. doi:https://doi.org/10.1016/0305-4179(95)00168-9

165. Fang Z, Li J, Wang K, et al. Autologous Scar-Related Tissue Combined with Skin Grafting for Reconstructing Large Area Burn Scar. *Journal of Investigative Surgery*. 2022;35(10):1779-1788. doi:10.1080/08941939.2022.2101164

166. Tredget EE, Shankowsky HA, Pannu R, et al. Transforming growth factor-beta in thermally injured patients with hypertrophic scars: effects of interferon alpha-2b. *Plast Reconstr Surg*. 1998;102(5):1317-1328; discussion 1329-30. doi:https://doi.org/10.1097/00006534-199810000-00001

167. Cheng W, Saing H, Zhou H, Han Y, Peh W, Tam PK. Ultrasound assessment of scald scars in Asian children receiving pressure garment therapy. *J Pediatr Surg*. 2001;36(3):466-469. doi:https://doi.org/10.1053/jpsu.2001.21613

168. Gokalp H. Evaluation of nonablative fractional laser treatment in scar reduction. *Lasers Med Sci*. 2017;32(7):1629-1635. doi:10.1007/s10103-017-2303-x

169. Abdullah A, Blakeney P, Hunt R, et al. Visible scars and self-esteem in pediatric patients with burns. *J Burn Care Rehabil*. 1994;15(2):164-168. doi:https://doi.org/10.1097/00004630-199403000-00011

170. Fisher I, Strong J, Tyack Z. Development, reliability, and concurrent validity of the modified inventory of potential reconstructive needs. *J Burn Care Rehabil*. 2001;22(2):154-162. doi:https://doi.org/10.1097/00004630-200103000-00013

171. *El Hoshy*.

172. Kwak IS, Young Park S, Choi YH, et al. Clinical and histopathological features of post burn pruritus. *Journal of Burn Care and Research*. 2016;37(6):343-349. doi:10.1097/BCR.0000000000000392

173. Wiseman J, Simons M, Kimble R, Ware RS, McPhail SM, Tyack Z. Effectiveness of topical silicone gel and pressure garment therapy for burn scar prevention and management in children 12-months postburn: A parallel group randomised controlled trial. *Clin Rehabil*. 2021;35(8):1126-1141. doi:10.1177/02692155211020351

174. Ge X, Sun Y, Lin J, Zhou F, Yao G, Su X. Effects of multiple modes of UltraPulse fractional CO2 laser treatment on extensive scarring: a retrospective study. *Lasers Med Sci*. 2022;37(3):1575-1582. doi:10.1007/s10103-021-03406-x

175. Van Der Wal MBA, Vloemans JFPM, Tuinebreijer WE, et al. Outcome after burns: An observational study on burn scar maturation and predictors for severe scarring. *Wound Repair and Regeneration*. 2012;20(5):676-687. doi:10.1111/j.1524-475X.2012.00820.x

176. Wang S, Mi J, Li Q, Jin R, Dong J. Fractional microplasma radiofrequency technology for non-hypertrophic post-burn scars in Asians: A prospective study of 95 patients. *Lasers Surg Med*. 2017;49(6):563-569. doi:10.1002/lsm.22640

177. Tawfic SO, Hassan AS, El-Zahraa Sh Aly F, Elbendary A, Shaker OG, AlOrbani AM. Fractional microneedle radiofrequency versus fractional carbon dioxide laser in the treatment of postburn hypertrophic scars. *Lasers Surg Med*. 2022;54(8):1089-1098. doi:https://doi.org/10.1002/lsm.23589

178. Erring M, Gaba S, Mohsina S, Tripathy S, Sharma RK. Comparison of efficacy of silver-nanoparticle gel, nano-silver-foam and collagen dressings in treatment of partial thickness burn wounds. *Burns*. 2019;45(8):1888-1894. doi:10.1016/j.burns.2019.07.019

179. Draaijers LJ, Botman YAM, Tempelman FRH, Kreis RW, Middelkoop E, Van Zuijlen PPM. Skin elasticity meter or subjective evaluation in scars: A reliability assessment. *Burns*. 2004;30(2):109-114. doi:10.1016/j.burns.2003.09.003

180. Jan SN, Bashir MM, Khan FA, et al. Unfiltered Nanofat Injections Rejuvenate Postburn Scars of Face. *Ann Plast Surg*. 2019;82(1):28-33. doi:10.1097/SAP.0000000000001631

181. Won T, Ma Q, Chen Z, Gao Z, Wu X, Zhang R. The efficacy and safety of low-energy carbon dioxide fractional laser use in the treatment of early-stage pediatric hypertrophic scars: A prospective, randomized, split-scar study. *Lasers Surg Med*. 2022;54(2):230-236. doi:https://doi.org/10.1002/lsm.23459

182. Müller B, Mazza E, Schiestl C, Elrod J. Longitudinal monitoring and prediction of long-term outcome of scar stiffness on pediatric patients. *Burns Trauma*. 2021;9. doi:10.1093/burnst/tkab028

183. Bloemen MCT, Van Gerven MS, Van Der Wal MBA, Verhaegen PDHM, Middelkoop E. An objective device for measuring surface roughness of skin and scars. *J Am Acad Dermatol*. 2011;64(4):706-715. doi:10.1016/j.jaad.2010.03.006

184. Goei H, van der Vlies CH, Tuinebreijer WE, van Zuijlen PPM, Middelkoop E, van Baar ME. Predictive validity of short term scar quality on final burn scar outcome using the Patient and Observer Scar Assessment Scale in patients with minor to moderate burn severity. *Burns*. 2017;43(4):715-723. doi:10.1016/j.burns.2016.10.012

185. Gee Kee EL, Kimble RM, Cuttle L, Stockton KA. Scar outcome of children with partial thickness burns: A 3 and 6 month follow up. *Burns*. 2016;42(1):97-103. doi:10.1016/j.burns.2015.06.019

186. Hjellestad M, Strand LI, Eide GE, Brekke R, Nesheim A, Gjelsvik BEB. Clinimetric properties of a translated and culturally adapted Norwegian version of the Patient and Observer Scar Assessment Scale for use in clinical practice and research. *Burns*. 2021;47(4):953-960. doi:10.1016/j.burns.2020.10.007

187. Klinger M, Caviggioli F, Klinger FM, et al. Autologous fat graft in scar treatment. In: *Journal of Craniofacial Surgery*. Vol 24. ; 2013:1610-1615. doi:10.1097/SCS.0b013e3182a24548

188. Simons M, Kimble R, McPhail S, Tyack Z. The Brisbane Burn Scar Impact Profile (child and young person version) for measuring health-related quality of life in children with burn scars: A longitudinal cohort study of reliability, validity and responsiveness. *Burns*. 2019;45(7):1537-1552. doi:10.1016/j.burns.2019.07.012

189. Simons M, Kimble R, McPhail S, Tyack Z. The longitudinal validity, reproducibility and responsiveness of the Brisbane Burn Scar Impact Profile (caregiver report for young children version) for measuring health-related quality of life in children with burn scars. *Burns*. 2019;45(8):1792-1809. doi:10.1016/j.burns.2019.04.015

190. Patel SP, Nguyen HV, Mannschreck D, Redett RJ, Puttgen KB, Stewart FD. Fractional CO 2 Laser Treatment Outcomes for Pediatric Hypertrophic Burn Scars. In: *Journal of Burn Care and Research*. Vol 40. Oxford University Press; 2019:386-391. doi:10.1093/jbcr/irz046

191. Spronk I, Stortelers A, van der Vlies CH, et al. Scar quality in children with burns 5–7 years after injury: A cross-sectional multicentre study. *Wound Repair and Regeneration*. 2021;29(6):951-960. doi:10.1111/wrr.12953

192. Van Der Wal MBA, Tuinebreijer WE, Bloemen MCT, Verhaegen PDHM, Middelkoop E, Van Zuijlen PPM. Rasch analysis of the Patient and Observer Scar Assessment Scale (POSAS) in burn scars. *Quality of Life Research*. 2012;21(1):13-23. doi:10.1007/s11136-011-9924-5

193. Wei Y, Wang Y, Zhang M, et al. The application of 3D-printed transparent facemask for facial scar management and its biomechanical rationale. *Burns*. 2018;44(2):453-461. doi:10.1016/j.burns.2017.08.006

194. Hundeshagen G, Collins VN, Wurzer P, et al. A prospective, randomized, controlled trial comparing the outpatient treatment of pediatric and adult partial-thickness burns with suprathel or Mepilex Ag. *Journal of Burn Care and Research*. 2018;39(2):261-267. doi:10.1097/BCR.0000000000000584

195. Ziolkowski NI, Behman R, Klassen AF, et al. Determining the Independent Risk Factors for Worse SCAR-Q Scores and Future Scar Revision Surgery. In: *Plastic and Reconstructive Surgery*. Vol 148. Lippincott Williams and Wilkins; 2021:203-212. doi:10.1097/PRS.0000000000008095

196. Busch K.H., Bender R., Walezko N., Aziz H., Altintas M.A., Aust M.C. COMBINATION OF MEDICAL NEEDLING AND NON-CULTURED AUTOLOGOUS SKIN CELL TRANSPLANTATION (RENOVACELL) FOR REPIGMENTATION OF HYPOPIGMENTED BURN SCARS IN CHILDREN AND YOUNG PEOPLE. *Ann Burns Fire Disasters*. 2016;29(2):116-122.

197. Van Der Wal M, Bloemen M, Verhaegen P, et al. Objective color measurements: Clinimetric performance of three devices on normal skin and scar tissue. *Journal of Burn Care and Research*. 2013;34(3). doi:10.1097/BCR.0b013e318264bf7d

198. van der Wal MBA, Tuinebreijer WE, Lundgren-Nilsson Å, Middelkoop E, van Zuijlen PPM. Differential item functioning in the Observer Scale of the POSAS for different scar types. *Qual Life Res*. 2014;23(7):2037-2045. doi:10.1007/s11136-014-0637-4

199. Liu XJ, Lei Y, Gold MH, Tan J. Efficacy of pulsed dye laser combined with fractional CO(2) laser in the treatment of pediatric burn scars. *Lasers Surg Med*. 2023;55(5):464-470. doi:https://doi.org/10.1002/lsm.23648

200. Kwa KA, Pijpe A, Korte D, Snoeks A, Breederveld RS, Meij-de Vries A. Using fibrin sealant for skin graft fixation to avoid sedation in children with burns: a prospective study. *J Wound Care*. 2020;29(11):642-648.

201. Miletta N, Siwy K, Hivnor C, et al. Fractional Ablative Laser Therapy is an Effective Treatment for Hypertrophic Burn Scars: A Prospective Study of Objective and Subjective Outcomes. *Ann Surg*. 2021;274(6):E574-E580. doi:10.1097/SLA.0000000000003576

202. Tatar BE. Investigating the Effects of Surgical Access Time on Scar Quality and Function in Pediatric Upper‑extremity Burn Contractures in a Rural Area. *Turkish Journal of Plastic Surgery*. 2024;32(1):27-31. doi:10.4103/tjps.tjps_60_23

203. Holmes JH, Molnar JA, Shupp JW, et al. Demonstration of the safety and effectiveness of the RECELL® System combined with split-thickness meshed autografts for the reduction of donor skin to treat mixed-depth burn injuries. *Burns*. 2019;45(4):772-782. doi:https://doi.org/10.1016/j.burns.2018.11.002

204. Simons M, Kee EG, Kimble R, Tyack Z. Ultrasound is a reproducible and valid tool for measuring scar height in children with burn scars: A cross-sectional study of the psychometric properties and utility of the ultrasound and 3D camera. *Burns*. 2017;43(5):993-1001. doi:10.1016/j.burns.2017.01.034

205. Barret JP, Dziewulski P, Jeschke MG, Wolf SE, Herndon DN. Effects of recombinant human growth hormone on the development of burn scarring. *Plast Reconstr Surg*. 1999;104(3):726-729. doi:https://doi.org/10.1097/00006534-199909030-00017

206. Branski LK, Herndon DN, Barrow RE, et al. Randomized Controlled Trial to Determine the Efficacy of Long-Term Growth Hormone Treatment in Severely Burned Children. *Ann Surg*. 2009;250(4):514-523. doi:https://doi.org/10.1097/sla06013e3181b819ca    https://doi.org/10.1097/sla.0b013e3181b8f9ca

207. Zuccaro J, Budd D, Kelly C, Fish JS. Pruritus in the Pediatric Burn Population. *JOURNAL OF BURN CARE & RESEARCH*. 2022;43(5):1175-1179. doi:https://doi.org/10.1093/jbcr/irac006

208. Branski LK, Herndon DN, Celis MM, Norbury WB, Masters OE, Jeschke MG. Amnion in the treatment of pediatric partial-thickness facial burns. *Burns*. 2008;34(3):393-399. doi:https://doi.org/10.1016/j.burns.2007.06.007

209. Branski LK, Herndon DN, Pereira C, et al. Longitudinal assessment of Integra in primary burn management: a randomized pediatric clinical trial. *Crit Care Med*. 2007;35(11):2615-2623. doi:https://doi.org/10.1097/01.ccm.0000285991.36698.e2

210. Crowe JM, Simpson K, Johnson W, Allen J. Reliability of photographic analysis in determining change in scar appearance. *J Burn Care Rehabil*. 1998;19(2):183-186. doi:https://doi.org/10.1097/00004630-199803000-00019

211. beausang1998-1.

212. Wang Q, Wang M, Xu Y, Ni XD, Cang ZQ, Yuan SM. Treatment of large scars in children using artificial dermis and scalp skin grafting. *Journal of Craniofacial Surgery*. 2019;30(3):891-896. doi:10.1097/SCS.0000000000005381

213. Keen A, Sheikh G, Hassan I, et al. Treatment of post-burn and post-traumatic atrophic scars with fractional CO2 laser: experience at a tertiary care centre. *Lasers Med Sci*. 2018;33(5):1039-1046. doi:10.1007/s10103-018-2469-x

214. Majid I, Imran S. Efficacy and safety of fractional CO 2 laser resurfacing in non-hypertrophic traumatic and burn scars . *J Cutan Aesthet Surg*. 2015;8(3):159. doi:10.4103/0974-2077.167276

215. Pan Y, Liang Z, Yuan S, Xu J, Wang J, Chen S. A long-term follow-up study of acellular dermal matrix with thin autograft in burns patients. *Ann Plast Surg*. 2011;67(4):346-351. doi:https://doi.org/10.1097/sap.0b013e3182111088

216. Kan T, Takahagi S, Matsubara D, et al. Clinical course of more than 10 years in a patient with extensive skin burns who received cultured epidermal autograft transplantation. *Regen Ther*. 2022;19:154-157. doi:https://doi.org/10.1016/j.reth.2022.01.007

217. Salles AG, Luitgards BF, Moraes LB, et al. Fractional carbon dioxide laser in patients with skin phototypes III to VI and facial burn sequelae: 1-year follow-up. *Plast Reconstr Surg*. 2018;142(3):342E-350E. doi:10.1097/PRS.0000000000004688

218. Clark JA, Leung’ KS, Chengz JCY, Leung’ PC. The hypertrophic scar and microcirculation. *Bums*. 1996;22(6):447-450.

219. Fraccalvieri M, Bogetti P, Salomone M, Di Santo C, Ruka E, Bruschi S. Cryotreatment of keloids: a single Italian institution experience. *Eur J Plast Surg*. 2016;39(3):201-206. doi:10.1007/s00238-015-1170-6

220. Wang LZ, Ding JP, Yang MY, Chen DW, Chen B. Treatment of facial post-burn hyperpigmentation using micro-plasma radiofrequency technology. *Lasers Med Sci*. 2015;30(1):241-245. doi:10.1007/s10103-014-1649-6

221. Maninder K, Richa R, Dinesh AP, Suman P. Factors affecting the outcome of fractional carbon dioxide laser resurfacing of various types of scars in skin of color. *J Cosmet Dermatol*. 2022;21(9):3842-3847. doi:10.1111/jocd.14698

222. Stiefel D, Schiestl C, Meuli M. Integra Artificial Skin® for burn scar revision in adolescents and children. *Burns*. 2010;36(1):114-120. doi:10.1016/j.burns.2009.02.023

223. Suzuki S, Kawai K, Ashoori F, Morimoto N, Nishimura Y, Ikada Y. Long-term follow-up study of artificial dermis composed of outer silicone layer and inner collagen sponge. *Br J Plast Surg*. 2000;53(8):659-666. doi:10.1054/bjps.2000.3426

224. Taki T, Kozuka S, Izawa Y, et al. Surgical Treatment of Skin Depigmentation Caused By Burn Injuries. *J Dermatol Surg Oncol*. 1985;11(12):1218-1221.

225. Trelles MA, Martínez-Carpio PA. Clinical and histological results in the treatment of atrophic and hypertrophic scars using a combined method of radiofrequency, ultrasound, and transepidermal drug delivery. *Int J Dermatol*. 2016;55(8):926-933. doi:10.1111/ijd.13253

226. Berry RB, Tan T, Cooke ED, et al. Transcutaneous oxygen tension as an index of maturity in hypet-trophic scars treated by compression. *Brirish Journal of Plastic Surgeons*. 1985;38:163-173.

227. Klöti J, Pochon JP. Conservative treatment using compression suits for second and third degree burns in children. *Burns*. 1982;8(3):180-187.

228. Kono T, Erçöçen AR, Nakazawa H, Honda T, Hayashi N, Nozaki M. The flashlamp-pumped pulsed dye laser (585 nm) treatment of hypertrophic scars in Asians. *Ann Plast Surg*. 2003;51(4):366-371. doi:10.1097/01.SAP.0000067722.07175.62

229. Lee SJ, Yeo IK, Kang JM, et al. Treatment of hypertrophic burn scars by combination laser-cision and pinhole method using a carbon dioxide laser. *Lasers Surg Med*. 2014;46(5):380-384. doi:10.1002/lsm.22247

230. Sawada Y, Sone K. *Treatment of Scars and Keloids with a Cream Containing Silicone Oil*. Vol 43.; 1990.

231. Tina S. Alster MD, Christopher A. Nanni MD. Pulsed Dye Laser Treatment of Hypertrophic Burn Scars. *Plast Reconstr Surg*. 1998;102(6):2190-2195.

232. Banov D, Banov F, Bassani AS. Case Series: The Effectiveness of Fatty Acids from Pracaxi Oil in a Topical Silicone Base for Scar and Wound Therapy. *Dermatol Ther (Heidelb)*. 2014;4(2):259-269. doi:10.1007/s13555-014-0065-y

233. Garcia Velasco.

234. Page RE, Barclay TL. Correlation of scald depth and hypertrophic scar formation. *Burns*. 1981;7(3):173-175. https://www.embase.com/search/results?subaction=viewrecord&id=L11178193&from=export

235. Pensler JM, Selvaggi TC, Parry SW. Facial resurfacing in the pediatric burn patient: a comparison of scar excision with dermabrasion. *J Burn Care Rehabil*. 1986;7(1):29-32. doi:https://doi.org/10.1097/00004630-198601000-00005

236. Romo EM, Fundora FP, Albajes CR, López LE, Hana Z. The effectiveness of cream with Centella Asiatica and Pinus Sylvestris to treat scars and burns. Clinical Trial. *Dermatologia Kliniczna*. 2012;14(3):105-110. https://www.embase.com/search/results?subaction=viewrecord&id=L368090618&from=export

237. Smith AC, Kimble R, Mill J, Bailey D, O’Rourke P, Wootton R. Diagnostic accuracy of and patient satisfaction with telemedicine for the follow-up of paediatric burns patients. *J Telemed Telecare*. 2004;10(4):193-198. doi:https://doi.org/10.1258/1357633041424449

238. Almaguer E, Dillon BT, Parry SW. Facial resurfacing at Shriners Burns Institute: a 16-year experience in young burned patients. *J Trauma*. 1985;25(11):1081-1082.

239. Basse P, Alsbj B, Lohmann M. The neck collar–a treatment of hypertrophic burn scars of the neck region. A clinical study. *Acta Chir Plast*. 1992;34(3):157-162.

240. Boyce ST, Goretsky MJ, Greenhalgh DG, Kagan RJ, Rieman MT, Warden GD. Comparative assessment of cultured skin substitutes and native skin autograft for treatment of full-thickness burns. *Ann Surg*. 1995;222(6):743-752. doi:https://doi.org/10.1097/00000658-199512000-00008

241. Erol OO, Gurlek A, Agaoglu G, Topcuoglu E, Oz H. Treatment of hypertrophic scars and keloids using intense pulsed light (IPL). *Aesthetic Plast Surg*. 2008;32(6):902-909. doi:https://doi.org/10.1007/s00266-008-9161-7

242. Lee SM, Ngim CK, Chan YY, Ho MJ. A comparison of Sil-K and Epiderm in scar management. *Burns*. 1996;22(6):483-487. doi:https://doi.org/10.1016/0305-4179(95)00165-4

243. Tymonová J, Adámková M, Klosová H, Kadlcík M, Zámecníková I. Our first experience with Integra. *Acta Chir Plast*. 2005;47(1):5-9.

244. Holmes JD, Rayner CR, Muir IF. The treatment of deep dermal burns by abrasion. *Scand J Plast Reconstr Surg Hand Surg*. 1987;21(3):237-240. doi:https://doi.org/10.3109/02844318709086449

245. Gunji H, Ono I, Tateshita T, Kaneko F. Clinical effectiveness of an ointment containing prostaglandin E1 for the treatment of burn wounds. *Burns*. 1996;22(5):399-405. doi:https://doi.org/10.1016/0305-4179(95)00170-0

246. Erol OO, Agaoglu G, Jawad MA. Combined Non-Ablative Laser and Microfat Grafting for Burn Scar Treatment. *Aesthet Surg J*. 2019;39(4):NP55-NP67. doi:https://doi.org/10.1093/asj/sjy291

247. Boyce ST, Kagan RJ, Yakuboff KP, et al. Cultured skin substitutes reduce donor skin harvesting for closure of excised, full-thickness burns. *Ann Surg*. 2002;235(2):269-279. doi:https://doi.org/10.1097/00000658-200202000-00016

248. Dolynchuk KN, Ziesmann M, Serletti JM. Topical putrescine (Fibrostat) in treatment of hypertrophic scars: phase II study. *Plast Reconstr Surg*. 1996;97(1):117-123; discussion 124-5. doi:https://doi.org/10.1097/00006534-199601000-00019

249. Duinslaeger L, Delaey B, Vanderkelen A. Short- and long-term results of application of allogeneic cultured keratinocytes on burn wounds and burn scar. *Eur J Plast Surg*. 1998;21(1):14-18. https://www.embase.com/search/results?subaction=viewrecord&id=L28077622&from=export

250. Floccard B, Tixier F, Chatot-Henry D, Lacotte B, Mehdaoui H, Drault JN. Early dermabrasion of deep dermal burns with sandpaper. Case reports. *Scand J Plast Reconstr Surg Hand Surg*. 1998;32(4):415-419. doi:https://doi.org/10.1080/02844319850158507

251. Raza MS, Asif MU, Abidin ZU, Khalid FA, Ilyas A, Tarar MN. Glycerol Preserved Amnion: A Viable Source of Biological Dressing for Superficial Partial Thickness Facial Burns. *J Coll Physicians Surg Pak*. 2020;30(4):394-398. doi:https://doi.org/10.29271/jcpsp.2020.04.394

252. Loren H Engrav M, Jourdan R Gottlieb M, Steven P Millard P, Marcus D Walkinshaw M, David M Heimbach M, Janet A Marvin RM. Partial excision of Residual Burn Lesions. *JBCR*. 1987;8(5):398-402.

253. Engrav LH, Gottlieb JR, Millard SP, Walkinshaw MD, Heimbach DM, Marvin JA. A comparison of intramarginal and extramarginal excision of hypertrophic burn scars. *Plast Reconstr Surg*. 1988;81(1):40-45.

254. Stern PJ, Law EJ, Benedict FE, MacMillan BG. Surgical treatment of elbow contractures in postburn children. *Plast Reconstr Surg* . 1985;76(3):441-446.

255. Kumagai N, Oshima H, Tanabe M, Ishida H, Uchikoshi T. Favorable donor site for epidermal cultivation for the treatment of burn scars with autologous cultured epithelium. *Ann Plast Surg*. 1997;38(5):506-513. doi:https://doi.org/10.1097/00000637-199705000-00011

256. Pham TN, Hanley C, Palmieri T, Greenhalgh DG. Results of early excision and full-thickness grafting of deep palm burns in children. *J Burn Care Rehabil*. 2001;22(1):54-57. doi:https://doi.org/10.1097/00004630-200101000-00011

257. Engrav LH, Heimbach DM, Reus JL, Harnar TJ, Marvin JA. Early excision and grafting vs. nonoperative treatment of burns of indeterminant depth: a randomized prospective study. *J Trauma*. 1983;23(11):1001-1004. doi:https://doi.org/10.1097/00005373-198311000-00007

258. Carney SA, Cason CG, Gowar JP, et al. *Cica-Care Gel Sheeting in the Management of Hypertrophic Scarring*. Vol 20.; 1994.

259. Fong SSL, Hung LK, Cheng JCY. The cutometer and ultrasonography in the assessment of postburn hypertrophic scar-a preliminary study. *Burns*. 1997;23(1):12.

260. Klöti J, Pochon JP. Long-term therapy of second and third degree burns in children using jobst-compression suits. *Scand J Plast Reconstr Surg Hand Surg*. 1979;13(1):163-166. doi:10.3109/02844317909013047

261. Kwon SD, Kye YC. Treatment of scars with a pulsed Er:YAG laser. *J Cutan Laser Ther*. 2000;2(1):27-31. doi:https://doi.org/10.1080/14628830050516579

262. Majani U, Majani A. Tissue mechanostimulation in the treatment of scars. *Acta Medica Mediterranea*. 2013;29(1):133-134. https://www.embase.com/search/results?subaction=viewrecord&id=L368961145&from=export

263. Murray TN, Guo EL, Richmond H, Friedman PM. Single treatment scar resurfacing with a novel ablative fractional 2910 nm erbium-doped fluoride glass fiber laser. *Lasers Surg Med*. 2024;56(1):19-22. doi:https://doi.org/10.1002/lsm.23729

264. Park GH, Rhee DY, Bak H, et al. Treatment of atrophic scars with fractional photothermolysis: short-term follow-up. *J Dermatolog Treat*. 2011;22(1):43-48. doi:https://doi.org/10.3109/09546630903473552

265. Ho WS, Chan HH, Ying SY, Chan PC, Burd A, King WWK. Prospective study on the treatment of postburn hyperpigmentation by intense pulsed light. *Lasers Surg Med*. 2003;32(1):42-45. doi:https://doi.org/10.1002/lsm.10139

266. E Beausang, H Floyd, K W Dunn, C I Orton, M W Ferguson. A new quantitative scale for clinical scar assessment. *Plast Reconstr Surg*. 1998;102(6):1954-1961.

267. Hirshowitz B, Lindenbaum E, Har-Shai Y, Feitelberg L, Tendler M, Katz D. Static-electric field induction by a silicone cushion for the treatment of hypertrophic and keloid scars. *Plast Reconstr Surg*. 1998;101(5):1173-1183.

268. Ohmori S, Tokyo J. Aesthetic Plasnc Surgery Effectiveness of Silastic Sheet Coverage in the Treatment of Scar Keloid (Hypertrophic Scar). *Aesth Plast Surg*. 1988;12:95-99.

269. Viani GA, Stefano EJ, Afonso SL, De Fendi LI. Postoperative Strontium-90 Brachytherapy in the Prevention of Keloids: Results and Prognostic Factors. *Int J Radiat Oncol Biol Phys*. 2009;73(5):1510-1516. doi:10.1016/j.ijrobp.2008.07.065

270. Madni TD, Nakonezny PA, Imran JB, et al. Patient satisfaction after fractional ablation of burn scar with 2940 nm wavelength Erbium-Yag laser. *Burns*. 2018;44(5):1100-1105. doi:10.1016/j.burns.2018.02.004

271. Herndon DN, LeMaster J, Beard S. The quality of life after major thermal injury in children: An analysis of 12 survivors with ≥ 80% total body, 70% third-degree burns. *Journal of Trauma*. 1986;26(7):609-619.

272. Ayaz M, Karami MY, Deilami I, Moradzadeh Z. Effects of Early Versus Delayed Excision and Grafting on Restoring the Functionality of Deep Burn-Injured Hands: A Double-Blind, Randomized Parallel Clinical Trial. *J Burn Care Res*. 2019;40(4):451-456. doi:https://doi.org/10.1093/jbcr/irz033

273. Frear CC, Cuttle L, McPhail SM, Chatfield MD, Kimble RM, Griffin BR. Randomized clinical trial of negative pressure wound therapy as an adjunctive treatment for small-area thermal burns in children. *British Journal of Surgery*. 2020;107(13):1741-1750. doi:10.1002/bjs.11993

274. Storey K, Lalloz M, Choy KT, et al. The versatility of biodegradable temporising matrix – A 63 paediatric case series with complex wounds. *Burns Open*. 2023;7(2):44-50. doi:https://doi.org/10.1016/j.burnso.2023.03.002

275. Mistry R, Issa F. No statistically significant difference in long term scarring outcomes of pediatric burns patients treated surgically vs. those treated conservatively. *Front Surg*. 2022;9. doi:10.3389/fsurg.2022.727983

276. Klassen AF, Ziolkowski N, Mundy LR, et al. Development of a new patient-reported outcome instrument to evaluate treatments for scars: The SCAR-Q. *Plast Reconstr Surg Glob Open*. 2018;6(4). doi:10.1097/GOX.0000000000001672

277. Xi W, Xie Y, Zhang Z, et al. 3D Mesh Releasing Method: A Retrospective Analysis of Fractional CO2 Treatment on Contracture Scars. *Lasers Surg Med*. 2021;53(2):227-235. doi:10.1002/lsm.23262

278. Wurzer P, Forbes AA, Hundeshagen G, et al. Two-year follow-up of outcomes related to scarring and distress in children with severe burns. *Disabil Rehabil*. 2017;39(16):1639-1643. doi:10.1080/09638288.2016.1209579

279. Zachariah JR, Lakshmanarao A, Prabha R, Gupta AK, Paul KM, Lamba S. A prospective study on the role of gabapentin in post-burn pruritus. *Eur J Plast Surg*. 2012;35(6):425-431. doi:https://doi.org/10.1007/s00238-011-0644-4

280. Rashaan ZM, Kwa KAA, van der Wal MBA, Tuinebreijer WE, van Zuijlen PPM, Breederveld RS. Patterns and predictors of burn scar outcome in the first 12 months after burn: The patient’s perspective. *Burns*. 2019;45(6):1283-1290. doi:10.1016/j.burns.2019.03.025

281. Fan C, Pek CH, Por YC, Lim GJS. Biobrane dressing for paediatric burns in Singapore: a retrospective review. *Singapore Med J*. 2018;59(7):360-365. doi:https://doi.org/10.11622/smedj.2017116

282. Loan F, Cassidy S, Marsh C, Simcock J. Keratin-based products for effective wound care management in superficial and partial thickness burns injuries. *Burns*. 2016;42(3):541-547. doi:https://doi.org/10.1016/j.burns.2015.10.024

283. Elrod J, Müller B, Christoph Mohr C, Meuli M, Mazza E, Schiestl C. An effective procedure for skin stiffness measurement to improve Paediatric Burn Care. *Burns*. 2019;45(5):1102-1111.

284. Cai LZ, Caceres M, Dangol MK, et al. Accuracy of remote burn scar evaluation via live video-conferencing technology. *Burns*. Published online 2016. doi:10.1016/j.burns.2016.11.006

285. Nolan MM, Reppucci ML, Urban A, et al. A Single Institution’s Recent Experience With Pediatric Hand Burns. *Journal of Burn Care and Research*. 2023;44(4):955-962. doi:10.1093/jbcr/irac174

286. Noureldin MA, Said TA, Makeen K, Kadry HM. Comparative study between skin micrografting (Meek technique) and meshed skin grafts in paediatric burns. *Burns*. 2022;48(7):1632-1644. doi:https://doi.org/10.1016/j.burns.2022.01.016

287. Rashaan ZM, Krijnen P, Allema JH, Vloemans AF, Schipper IB, Breederveld RS. Usability and effectiveness of Suprathel® in partial thickness burns in children. *European Journal of Trauma and Emergency Surgery*. 2017;43(4):549-556. doi:10.1007/s00068-016-0708-z

288. Ziolkowski NI, Pusic AL, Fish JS, et al. Psychometric Findings for the SCAR-Q Patient-Reported Outcome Measure Based on 731 Children and Adults with Surgical, Traumatic, and Burn Scars from Four Countries. *Plast Reconstr Surg*. 2020;146(3):331E-338E. doi:10.1097/PRS.0000000000007078

289. Wiseman J, Simons M, Kimble R, Tyack Z. Variability of pressure at the pressure garment-scar interface in children after burn: A pilot longitudinal cohort study. *Burns*. 2019;45(1):103-113. doi:10.1016/j.burns.2018.08.029

290. Ren J, Liu J, Yu N, et al. The use of noncultured regenerative epithelial suspension for improving skin color and scars: A report of 8 cases and review of the literature. *J Cosmet Dermatol*. 2019;18(5):1487-1494. doi:https://doi.org/10.1111/jocd.13071

291. Rogge FJ, Cambier B. Safe and effective treatment of problem scars with the purely thermal non-ablative Er:YAG laser scar mode. *Journal of Cosmetic and Laser Therapy*. 2008;10(3):143-147. doi:10.1080/14764170802132694

292. Chan HH, Wong DSY, Ho WS, Lam LK, Wei AW. The Use of Pulsed Dye Laser for the Prevention and Treatment of Hypertrophic Scars in Chinese Persons. *Dermatologic surgery : official publication for American Society for Dermatologic Surgery*. 2004;30(7):987-994.

293. Donnellan KA, Hinson CS, Blevins AC, et al. Treatment of Chronic Hypertrophic Burn Scars With a Fractional CO2Laser Is Well Tolerated in an Outpatient Clinic Setting. *Ann Plast Surg*. 2023;90(5):444-446. doi:10.1097/SAP.0000000000003526

294. den Kerchhove E, Boeckx W, Kochuyt A. Silicone patches as a supplement for pressure therapy to control hypertrophic scarring. *J Burn Care Rehabil*. 1991;12(4):361-369. doi:https://doi.org/10.1097/00004630-199107000-00015

295. Hultman CS, Friedstat JS, Edkins RE. Efficacy of intense pulsed light for the treatment of burn scar dyschromias: a pilot study to assess patient satisfaction, safety, and willingness to pay. *Ann Plast Surg*. 2015;74:S204-S208. doi:https://doi.org/10.1097/sap.0000000000000447

296. Engrav LH, Heimbach DM, Rivara FP, et al. 12-Year within-wound study of the effectiveness of custom pressure garment therapy. *Burns*. 2010;36(7):975-983. doi:10.1016/j.burns.2010.04.014

297. Wei Y, Li-Tsang CWP, Liu J, Xie L, Yue S. 3D-printed transparent facemasks in the treatment of facial hypertrophic scars of young children with burns. *Burns*. 2017;43(3):e19-e26. doi:10.1016/j.burns.2016.08.034

298. Kim JD, Oh SJ, Kim SG, et al. Ultrasonographic findings of re-epithelialized skin after partial-thickness burns. *Burns Trauma*. 2018;6(1):21. doi:https://doi.org/10.1186/s41038-018-0122-3

299. Yim H, Cho YS, Seo CH, et al. The use of AlloDerm on major burn patients: AlloDerm prevents post-burn joint contracture. *Burns*. 2010;36(3):322-328. doi:https://doi.org/10.1016/j.burns.2009.10.018

300. Wang XQ, Mill J, Kravchuk O, Kimble RM. Ultrasound assessed thickness of burn scars in association with laser Doppler imaging determined depth of burns in paediatric patients. *Burns*. 2010;36(8):1254-1262. doi:https://doi.org/10.1016/j.burns.2010.05.018

301. Di Santolo MS, Sagnelli M, Tortora G, et al. The utility of the high‑resolution ultrasound technique in the evaluation of autologous adipose tissue lipofilling, used for the correction of post‑surgical, post‑traumatic and post‑burn scars. *Radiologia Medica*. 2016;121(6):521-527. doi:10.1007/s11547-016-0621-x

302. Peeters W, Anthonissen M, Deliaert A, der Hulst R, den Kerckhove E. A comparison between laser-doppler imaging and colorimetry in the assessment of scarring: “a pilot study.” *Skin Res Technol*. 2012;18(2):188-191. doi:https://doi.org/10.1111/j.1600-0846.2011.00552.x

303. Li-Tsang CW, Lau JC, Liu SK. Validation of an objective scar pigmentation measurement by using a spectrocolorimeter. *Burns*. 2003;29(8):779-784. doi:https://doi.org/10.1016/s0305-4179(03)00165-7

304. Rahmanian-Schwarz A, Beiderwieden A, Willkomm LM, Amr A, Schaller HE, Lotter O. A clinical evaluation of Biobrane(®) and Suprathel(®) in acute burns and reconstructive surgery. *Burns*. 2011;37(8):1343-1348. doi:https://doi.org/10.1016/j.burns.2011.07.010

305. Matsuzaki K, Kumagai N, Fukushi S, Ohshima H, Tanabe M, Ishida H. Cultured epithelial autografting on meshed skin graft scars: evaluation of skin elasticity. *J Burn Care Rehabil*. 1995;16(5):496-502. doi:https://doi.org/10.1097/00004630-199509000-00006

306. Quinn KJ. Silicone gel in scar treatment. *Burns Incl Therm Inj*. 1987;13:S33-S40. doi:https://doi.org/10.1016/0305-4179(87)90091-x

307. Karwacińska J, Kiebzak W, Stepanek-Finda B, et al. Effectiveness of kinesio taping on hypertrophic scars, keloids and scar contractures. *Polish Annals of Medicine*. 2012;19(1):50-57. doi:10.1016/j.poamed.2012.04.010

308. Haq MA, Haq A. Pressure therapy in treatment of hypertrophic scar, burn contracture and keloid: the Kenyan experience. *East Afr Med J*. 1990;67(11):785-793.

309. Hambleton J, Shakespeare PG, Pratt BJ. The progress of hypertrophic scars monitored by ultrasound measurements of thickness. 1992;18(4):301-307.

310. Sadove AM, Jones JE, Lynch TR, Sheets PW. Appliance therapy for perioral electrical burns: a conservative approach. *J Burn Care Rehabil*. 1988;9(4):391-395. doi:https://doi.org/10.1097/00004630-198807000-00015

311. RUSCIANI L, ROSSI G, BONO R. Use of Cryotherapy in the Treatment of Keloids. *J Dermatol Surg Oncol*. 1993;19(6):529-534. doi:10.1111/j.1524-4725.1993.tb00386.x

312. Wang ZY, Zhang J, Lu SL. Objective evaluation of burn and post-surgical scars and the accuracy of subjective scar type judgment. *Chin Med J (Engl)*. 2008;121(24):2517-2520. doi:https://doi.org/10.1097/00029330-200812020-00009

313. Sawada Y. A method of recording and objective assessment of hypertrophic burn scars. *Burns*. 1994;20(1):76-78. doi:https://doi.org/10.1016/0305-4179(94)90113-9

314. J.P. Shepherd, B.D.S. M.Sc., F.D.S.R.C.S., R.P.R. Dawber, M.A., F.R.C.P. The Response of Keloid Scars to Cryosurgery. *Plast Reconstr Surg*. 1982;70(6):677-681.

315. Stekelenburg CM, Simons JM, Tuinebreijer WE, van Zuijlen PPM. Analyzing contraction of full thickness skin grafts in time: Choosing the donor site does matter. *Burns*. 2016;42(7):1471-1476. doi:10.1016/j.burns.2016.02.001

316. Stekelenburg CM, Van Der Wal MBA, Knol DL, De Vet HCW, Van Zuijlen PPM. Three-dimensional digital stereophotogrammetry: A reliable and valid technique for measuring scar surface area. *Plast Reconstr Surg*. 2013;132(1):204-211. doi:10.1097/PRS.0b013e318290f675

317. Peake M, Pan K, Rotatori RM, et al. Incorporation of 3D stereophotogrammetry as a reliable method for assessing scar volume in standard clinical practice. *Burns*. 2019;45(7):1614-1620. doi:10.1016/j.burns.2019.05.005

318. H. Paul Ehrlich PhD, Sean F. Kelley BS. Hypertrophic Scar: An Interruption in the Remodeling of Repair - A Laser Doppler Blood Flow Study. *Plast Reconstr Surg*. 1992;90(6):993-998.

319. Leung KS, Sher A, Clark JA, Cheng JC, Leung PC. Microcirculation in hypertrophic scars after burn injury. *J Burn Care Rehabil*. 1989;10(5):436-444. doi:https://doi.org/10.1097/00004630-198909000-00013
